# Branched Fluorenylidene Derivatives with Low Ionization Potentials as Hole-Transporting Materials for Perovskite Solar Cells

**DOI:** 10.1021/acs.chemmater.3c00708

**Published:** 2023-07-29

**Authors:** Aistė Jegorovė, Jianxing Xia, Matas Steponaitis, Maryte Daskeviciene, Vygintas Jankauskas, Alytis Gruodis, Egidijus Kamarauskas, Tadas Malinauskas, Kasparas Rakstys, Khalid A. Alamry, Vytautas Getautis, Mohammad Khaja Nazeeruddin

**Affiliations:** ‡ Department of Organic Chemistry, Kaunas University of Technology, Radvilenu pl. 19, Kaunas, 50254 Lithuania; ∥ Institute of Chemical Sciences and Engineering, École Polytechnique Federale de Lausanne (EPFL), Lausanne, 1015 Switzerland; ∇ Institute of Chemical Physics, Vilnius University, Sauletekio al. 3, Vilnius, 10257 Lithuania; § Chemistry Department, Faculty of Science, King Abdulaziz University, P.O. Box 80203, 21589 Jeddah, Saudi Arabia

## Abstract

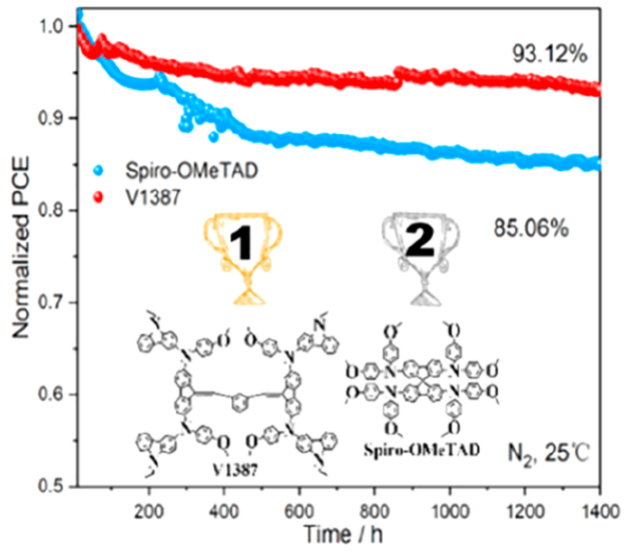

A group of small-molecule hole-transporting materials (HTMs) that are based on fluorenylidene fragments were synthesized and tested in perovskite solar cells (PSCs). The investigated compounds were synthesized by a facile two-step synthesis, and their properties were measured using thermoanalytical, optoelectronic, and photovoltaic methods. The champion PSC device that was doped with lithium bis(trifluoromethanesulfonyl)imide (LiTFSI) reached a power conversion efficiency of 22.83%. The longevity of the PSC device with the best performing HTM, **V1387**, was evaluated in different conditions and compared to that of 2,2′,7,7′-tetrakis(*N,N*-di-*p*-methoxyphenylamine)-9,9′-spirobifluorene (spiro-MeOTAD), showing improved stability. This work provides an alternative HTM strategy for fabricating efficient and stable PSCs.

## 1 Introduction

Since the naming of the first perovskite in the 19th century,^1^ various materials have been discovered and described as perovskites.^2−4^ These materials can be organic,^5^ inorganic,^6^ or hybrid.^7^ With a few exceptions, perovskites are generalized with the formula ABX_3_, where A and B are cations of different charges (mono- and dications) and X is an anion that coordinates B. The recent attention of perovskite materials for use in optoelectronics should come as no surprise, as they have large absorption coefficients, tunable compositions and absorption edges, long charge carrier diffusion lengths, high defect tolerances, and efficient charge transport properties.^8,9^ Furthermore, perovskites can be processed in a variety of different ways,^10−13^ making them attractive not only for research but also for potential commercial applications.^14,15^

One of the most researched topics in science is the study of perovskite solar cells (PSCs). Since the first article published in 2009, the efficiency of PSCs has skyrocketed from 3.8% to over 26%.^16^ However, the rapid emergence of PSCs does not mean the technology has no flaws. Unsolved fabrication issues, unstable charge-transporting materials, and the long-term stability of perovskite compositions and devices must be addressed before this technology can reach the market. Additionally, using a quality hole-transporting material (HTM) is crucial for creating efficient and stable PSC devices.^17,18^ The function of an HTM is to efficiently transport photogenerated positive carriers from the absorber to the electrode. To achieve this, the HTM has to be chemically and morphologically stable, have the appropriate energy levels, and have a relatively high carrier mobility.^19^ Over the years, efficiency records have mostly been broken by devices using either 2,2′,7,7′-tetrakis(*N,N*-di-*p*-methoxyphenylamine)-9,9′-spirobifluorene (spiro-MeOTAD) or poly[bis(4-phenyl)(2,4,6-trimethylphenyl)amine] (PTAA) as an HTM. However, these materials are expensive and relatively difficult to synthesize, and spiro-MeOTAD is known to have stability issues.^20−23^ In order to replace spiro-MeOTAD, researchers have synthesized many new molecules with the same “award winning” structural design,^24−26,26^ hoping to achieve higher power conversion efficiencies (PCEs). Various polymers have also been developed in an attempt to replace PTAA as an HTM.^27−30^ To mimic spiro-MeOTAD, there are two commonly used strategies: (1) keep the central spiro core and only change the substituents around it^31−33^ or (2) build a molecule by carefully choosing substituents around a chosen central core in order to imitate the spatial arrangement of spiro-MeOTAD.^34−36^ Early on in the research of PSCs, the first strategy proved more fruitful, successfully creating PSCs with higher PCEs. The second strategy then became more popular later on, when the cost and stability of PSC devices became factors that needed to be addressed.

In one of our previous works, we demonstrated that using HTMs based on methoxydiphenylamine-substituted fluorene derivatives with a small central core is a good strategy for fabricating highly efficient PSCs.^37^ To expand on our aforementioned study, we decided to employ carbazole-containing substituents as electron donating units, as this is known to tune the highest occupied molecular orbital (HOMO) level and help reach a high PCE.^38−40^ Furthermore, carbazole is a suitable building block for HTMs due to the possibility of having numerous substitutions of the carbazole unit. Various carbazole-containing scaffolds as electron donating units were used in order to tune the energy levels of HTMs, showing a good photovoltaic performance. For example, SGT series,^41,42^ benzodithiazole-,^43^ bipyridine-,^44^ and pyrene-based^45^ electron donating units were used to fabricate highly efficient devices.

In this work, we describe the synthesis and application of new HTMs comprised of various central core units and substituted carbazole derivatives. These materials can be obtained in a facile two-step synthesis procedure. Their thermal, optical, and photoelectrical properties were also thoroughly investigated. All of the tested novel HTMs were successfully applied in PSCs, reaching an efficiency of up to 22%. Furthermore, the device employing the best performing HTM, **V1387**, demonstrated improved long-term stability compared to PSCs that use spiro-MeOTAD as a *p*-type charge carrier.

## 2 Results and Discussion

**Scheme 1:**
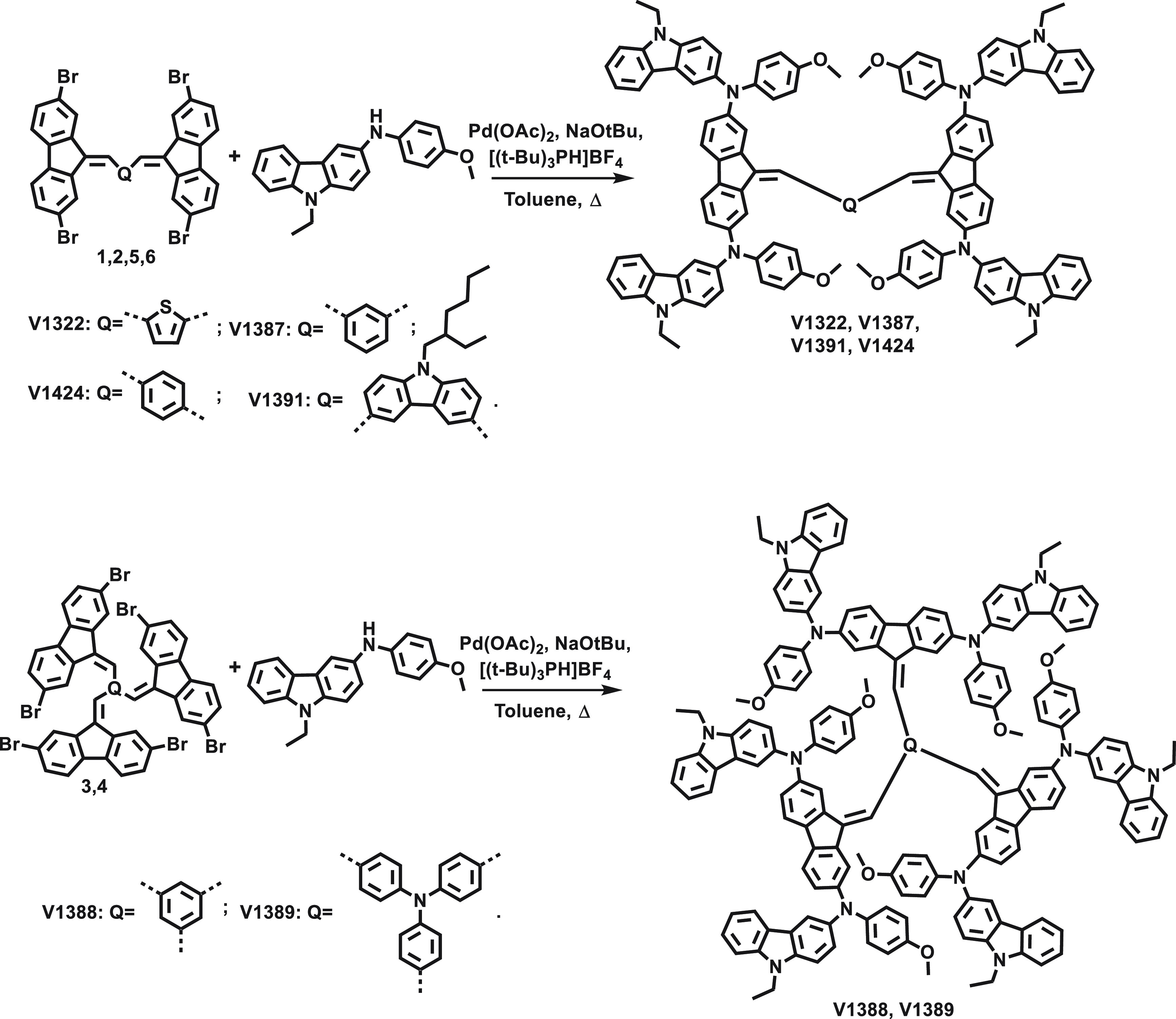
Synthesis of the HTMs **V1322**, **V1387**, **V1391**, **V1424**, **V1388**, and **V1389**

All of the newly synthesized HTMs can be divided into two groups: molecules with two fluorene units in their central core structure and molecules with three fluorene units. Compounds **1**–**6** were synthesized by a base-catalyzed condensation reaction; an example of said reaction can be seen in Scheme S1. All HTMs were obtained by palladium cross-coupling reactions between the respective central core unit and 9-ethyl-*N*-(4-metoxyphenyl)-9*H*-carbazol-3-amine (Scheme 1). Detailed synthetic protocols of said materials are described in the Supporting Information.

**Figure 1:**
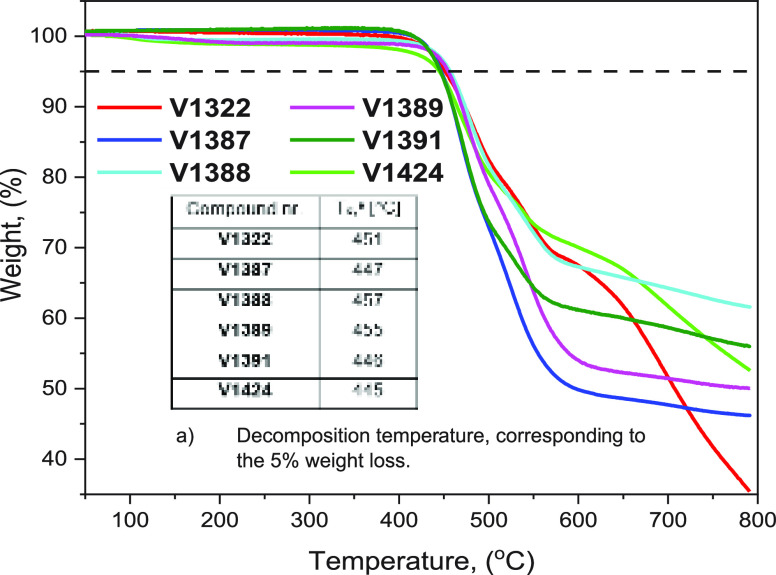
TGA curves of the synthesized HTMs.

To determine the thermal and morphological stability of the HTMs, thermogravimetric analysis (TGA) and differential scanning calorimetry (DSC) were used. Analysis of the TGA data suggests that all HTMs decompose around 450 °C (Figure 1), far above the temperature required for conventional device operation.^14^ DSC measurements reveal that the new organic semiconductors are molecular glasses having glass transition temperatures (*T*_g_) between 190 and 232 °C (Figure S1), surpassing the *T*_g_ of spiro-MeOTAD (124 °C).^46^ This suggests that our new materials are more morphologically stable.

Quantum chemistry simulations of the ground state molecular structures for several of the most probable conformers were provided using Gaussian 16^47^ software by means of density functional theory (DFT) via the B3LYP method and a 6-31G(d) basis set, supplemented with polarization functions (d). Solvation effects were not considered in all cases. A list of several of the most probable molecular conformations is presented in Table S2. Two projections (*xy* and *xz*) of each of the mentioned conformations are presented in Figures S5–S10. In all cases, it is necessary to point out that the molecular structures are presented in a chaotic manner due to the absence of total or partial symmetry and the existence of a very large number of different conformers. All presented structures in Table S2 were obtained using a gradient optimization technique (convergence of the maximum force, root mean square (RMS) force, maximum displacement, and RMS displacement parameters has been achieved). Electronic excitations were simulated using a semi-empirical temporal difference (TD) method (for singlets). The parameters of the electronic excitations (transition energies Δ*E*_1_ (S_0_ → S_1_) and Δ*E*_2_ (S_0_ → S_2_) and corresponding oscillator strengths *f*_1_ and *f*_2_) are presented in Table S7. The populations of the low lying excited molecular states S_1_ and S_2_ could be realized using forbidden transitions: S_0_ → S_*n*_, where *n* = 1 and 2 (oscillator strengths *f_n_* → 0). In a condensed phase, due to close intermolecular contacts, this prohibition is partially removed. The parameters of the transition between the molecular orbitals (MOs), which are related to the population of the “spectroscopic” states S_*n*_, (*n* = 1 and 2) are presented in Table S3. In all cases, the dominant and most significant electron jump exists between the HOMO and the lowest unoccupied molecular orbital (LUMO). The spatial distributions of electron density (for the HOMO–1, HOMO, LUMO, and LUMO+1 of each compound) are presented in Tables S4–S6. For **V1322**, the thiophene core plays an important role in establishing the charge redistribution between the center core (and partially the left substituent) and the right substituent (see Table S4). For **V1387**, the phenyl core (linked in positions 1 and 3 to two fluorene substituents) forms a unit that has a similar role: to establish the charge redistribution between the left and right substituents and the central core (see Table S4). For **V1391**, a central core is formed using carbazole with two linked fluorenes, and a core fragment takes part in the charge redistribution between the left and right substituents and the central core (see Table S5). For **V1424**, the phenyl core (linked in positions 1 and 4 to two fluorene substituents) forms a unit that has a similar role: to establish the charge redistribution between the left and right substituents and the central core (see Table S5). The formation of a central unit is more effective for **V1424** than in the case of **V1387**, due to the different link conditions. For **V1388**, the phenyl core (linked in positions 1, 3, and 5 to three fluorene substituents) forms a unit for establishing the charge redistribution between the left and right substituents and the central core (see Table S6). For **V1389**, a central unit is formed from triphenylamine (TPA), and the charge redistribution between the left and right substituents and the central core is established (see Table S6).

**Figure 2:**
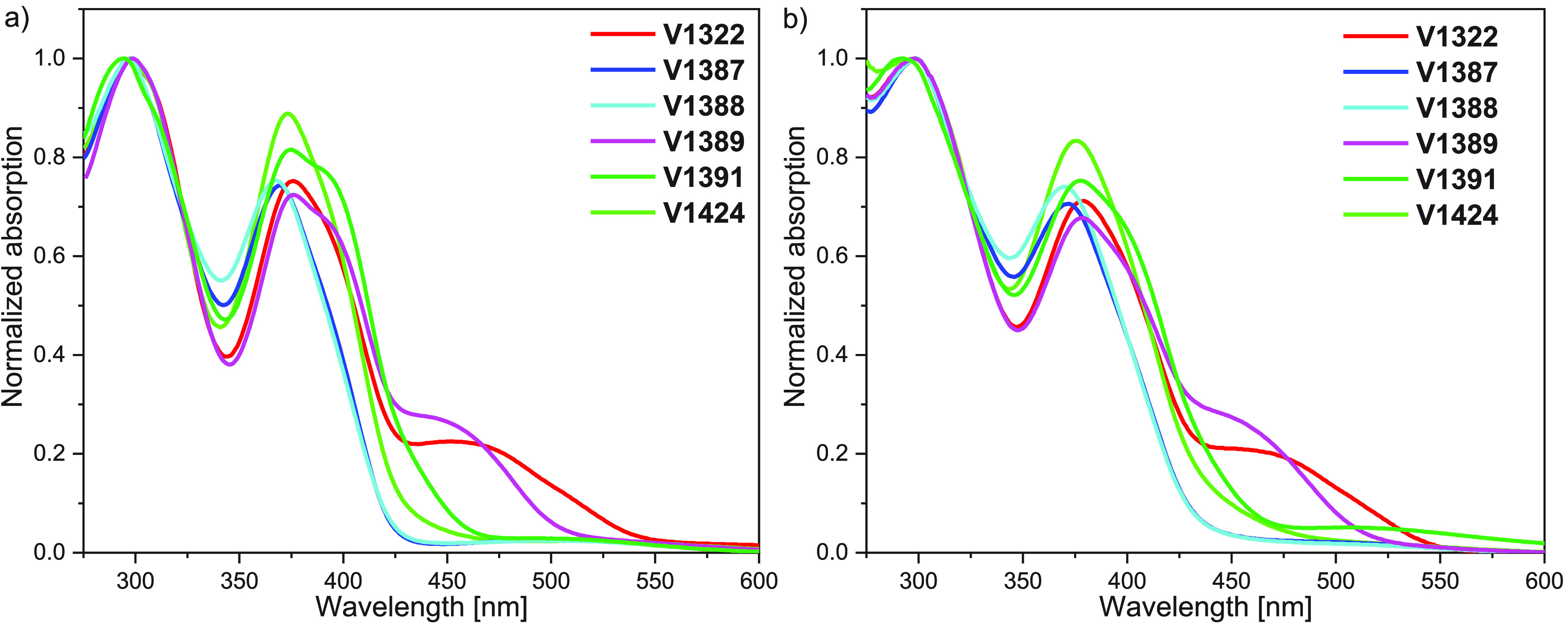
UV–vis absorption spectra of the V series HTMs (a) in a THF solution and (b) as thin films on glass substrates.

The ultraviolet–visible (UV–vis) absorption spectra of the V series HTMs were measured in a tetrahydrofuran (THF) solution and on glass substrates (Figure 2a,b). All of the compounds have two major absorption peaks at around 300 and 375 nm, which represent the π–π* transitions. The materials **V1322** and **V1389** both have an extra peak in the 450–470 nm range, which most likely corresponds to the π–π* transitions with some charge transfer character, owing to the electron-rich nature of triarylamine and thiophene π systems. When comparing the spectra of the same compounds in solution to those acting as thin films, practically no shift in absorption is observed.

**Figure 3:**
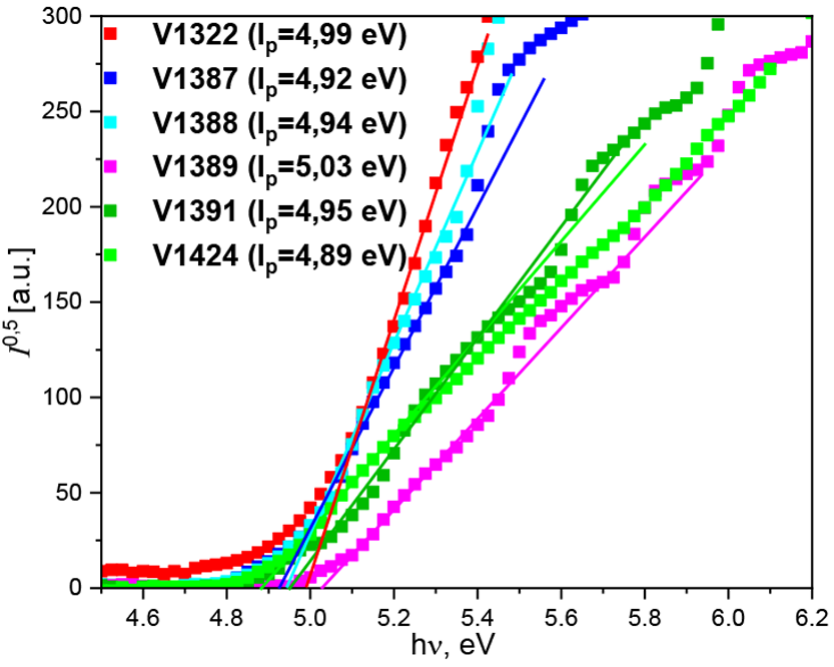
Ionization potential of the compounds **V1322**, **V1387**, **V1388**, **V1389**, **V1391**, and **V1424**.

To evaluate the energy levels of the synthesized materials in the solid state, the ionization potential (*I*_p_) was measured by photoelectron spectroscopy in air (PESA), where the measurement error was evaluated to be 0.03 eV. The *I*_p_ values of the synthesized HTMs can be seen in Figure 3. All of the tested materials have a relatively high HOMO energy level at ∼5.0 eV. After analyzing the data, it seems that the biggest influence on *I*_p_ is the central core rather than the number of substitutions around it. The HTMs that contain single phenyl rings in their core (**V1387**, **V1388**, and **V1424**) have the highest HOMO levels, while the HTMs that have carbazole, thiophene, and TPA central fragments have slightly lower energy levels. This minor difference in *I*_p_ could be the cause of slightly bulkier cores, which in turn causes more steric hindrance in the solid state.

**Figure 4:**
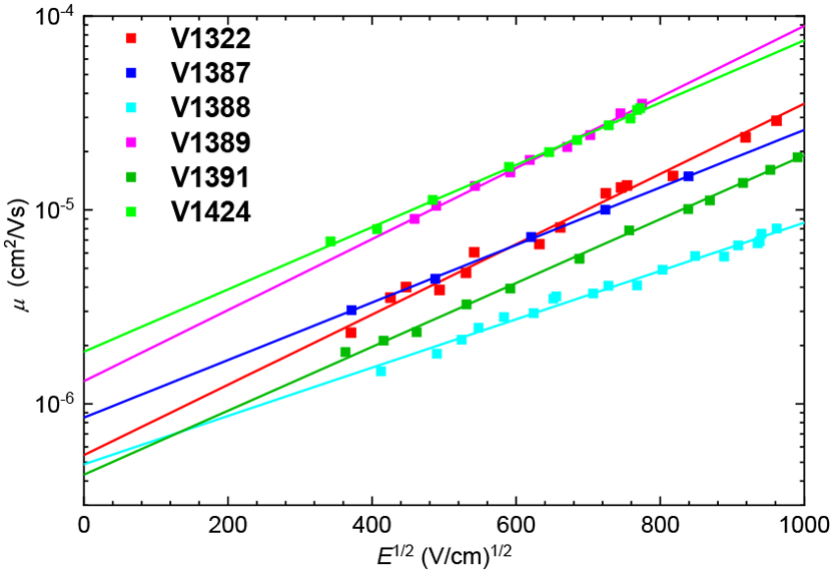
Charge carrier mobility of the V series HTMs, measured with PC-Z.

Hole mobility was measured from thin films via the xerographic time-of-flight (XTOF) method, with the electric field dependencies of the hole drift mobilities shown in Figure 4. Due to poor layer formation, the investigated materials had to be mixed with bisphenol Z polycarbonate (PC-Z, weight ratio 1:1) in order to form uniform thin films, which are necessary for accurate drift carrier mobility evaluation. **V1424** had the highest zero-field hole mobility (*μ*_0_ = 1.9 × 10^–6^ cm^2^ V^–1^ s^–1^), while the mobility values for the other semiconductors were slightly lower. In comparison to spiro-MeOTAD (*μ*_0_ = 1.3 × 10^–4^ cm^2^ V^–1^ s^–1^),^48^ these values are significantly lower. However, it is worth noting that results acquired from HTM mixtures with PC-Z are generally at least one order of magnitude lower than those of the pristine material. The thermal, optical, and photoelectrical properties of the new *p*-type semiconductors are summarized in Table 1.

**Table 1:** Thermal, Optical, and Photophysical Properties of the Synthesized Materials

ID	*T*_g_ (°C)^a^	*T*_dec_ (°C)^a^	*λ*_abs_ (nm)^b^	*λ*_abs_ (nm)^c^	*I*_p_ (eV)^d^	*E*_g_ (eV)^e^	*μ*_0_ (cm^2^ V^–1^ s^–1^)^f^
**V1322**	190	451	298, 376, 461	299, 379, 466	4.99	2.25	5.4 × 10^–7^
**V1387**	209	452	297, 369	299, 372	4.92	2.82	8.5 × 10^–7^
**V1388**	210	457	297, 367	299, 370	4.94	2.82	4.9 × 10^–7^
**V1389**	231	455	299, 376, 451	298, 379, 454	5.03	2.38	1.3 × 10^–6^
**V1391**	206	446	295, 375	293, 378	4.95	2.59	4.3 × 10^–7^
**V1424**	203	445	298, 373	298, 375	4.89	2.64	1.9 × 10^–6^

a The glass transition (*T*_g_) and decomposition (*T*_dec_) temperatures were determined by DSC and TGA, respectively (10 °C/min, N_2_ atmosphere).

b The absorption spectra were measured in a THF solution (10^–4^ M).

c The absorption spectra were measured from thin films on glass substrates.

d The HOMO energy levels of the thin films were measured using PESA.

e The optical band gaps *E*_g_ were estimated from the edges of the electronic absorption spectra in the solid state.

f The mobility values at zero field strength.

**Figure 5:**
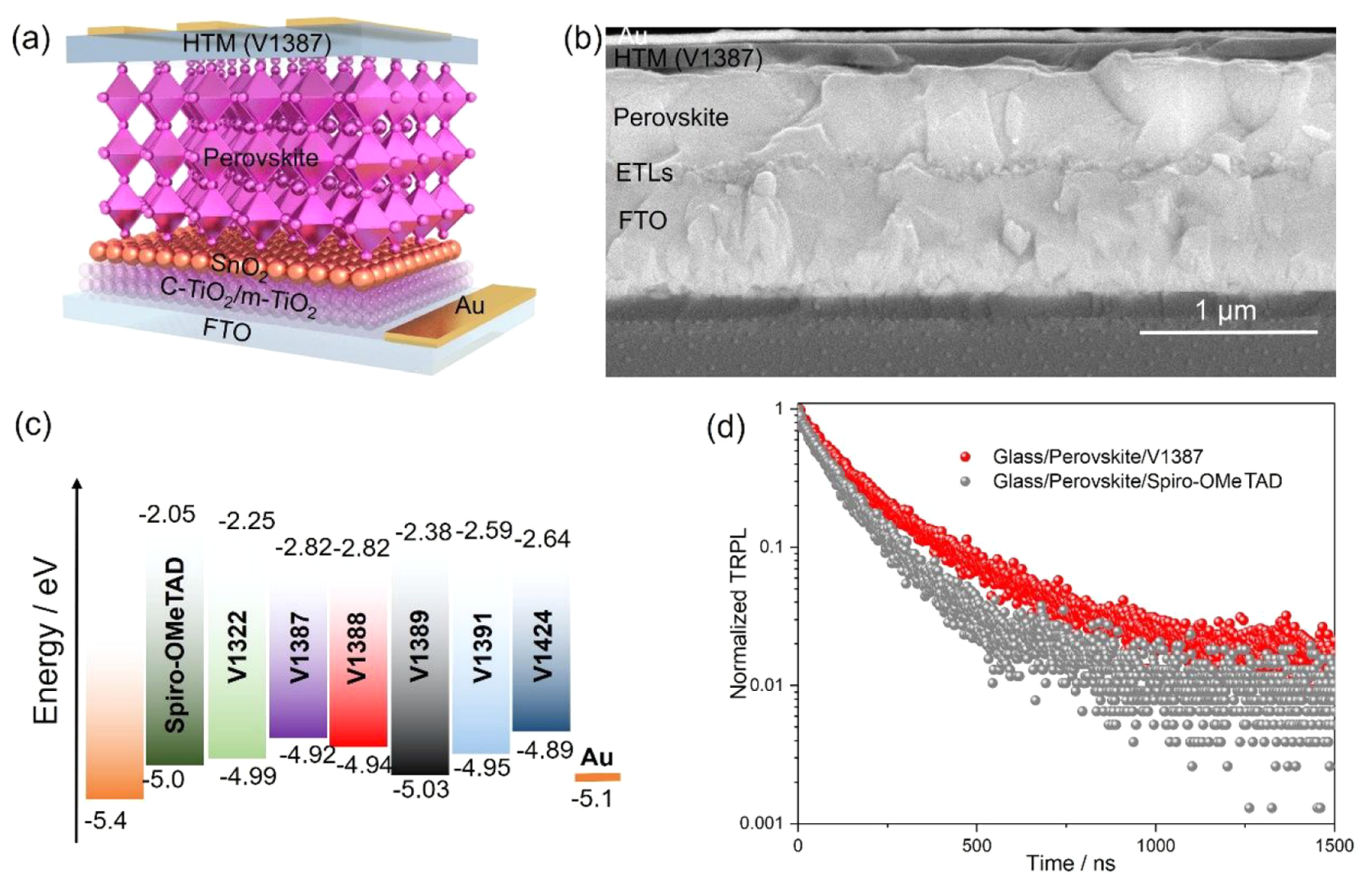
(a) A representative illustration of the structure of the fabricated PSCs. (b) A cross-sectional SEM image of the solar cell that used **V1387** as its HTM. (c) The energy levels of the various HTMs created in this study compared against sprio-OMeTAD and gold. (d) The TRPL measurements of a sample based on glass/perovskite/**V1387** (red) and of a sample based on glass/perovskite/spiro-OMeTAD (gray).

Next, PSCs were prepared with the synthesized materials in a regular configuration (*n*-*i*-*p*). For each PSC, fluorinated tin oxide (FTO) was used as a front contact, SnO_2_ and compact and mesoporous TiO_2_ (c-TiO_2_ and m-TiO_2_, respectively) were used as the electron-transporting layers (ETLs), a triple cation perovskite was used as the light absorber, and the synthesized HTM was responsible for transporting positive charges toward the back contact, which in this case was gold (Figure 5a). Cross-sectional scanning electron microscopy (SEM) images of one of the devices can be seen in Figure 5b. Surface SEM was also used to study **V1387** on the perovskite layer. As compared with the bare perovskite film, the additional layer of **V1387** fully covered the perovskite crystal (Figure S2). The energy level illustration of various HTMs depicted in Figure 5c shows that all of the HTMs that were created in this study have around the same energy as spiro-OMeTAD; thus, they are suitable for hole transportation in PSCs.^49,50^ Time-resolved photoluminescence (TRPL, Figure 5d) was performed on the glass/perovskite/**V1387** and glass/perovskite/spiro-OMeTAD films. The TRPL decay time was fitted by a bi-exponential model with fast (τ_1_) and slow (τ_2_) components, which indicate the interfacial transportation and recombination, respectively. The average decay time (τ_ave_) is calculated using the equation *τ*_ave_ = ∑*A*_*i*_*τ*_*i*_^2^/∑*A*_*i*_*τ*_*i*_, where *A*_*i*_ and *τ*_*i*_ represent the decay amplitude and the decay time component, respectively (Table 2). For interfacial transport, the fast (τ_1_) component was considered. When the spiro-OMeTAD was employed for the thin film, the decay time was 68.7 ns, with an average decay time of 143.4 ns. In comparison, for the perovskite/**V1387** interface, a slightly slower decay time with *τ*_1_ = 82.8 ns and an average decay time of τ = 227.5 ns were obtained. These results imply that **V1387**-based PSCs would exhibit a slightly lower or similar photovoltaic performance compared to that of spiro-OMeTAD-based PSCs.

**Table 2:** Fitting Parameters of the Bi-Exponential Decay Function for TRPL Analysis of Glass/Perovskite Samples with Spiro-OMeTAD and **V1387**

films	fraction *A*_1_ (%)	τ_1_ (ns)	fraction *A*_2_ (%)	τ_2_ (ns)	average decay time, τ_ave_ (ns)^a^
FTO/perovskite/spiro-OMeTAD	61.1	68.7	38.9	186.7	143.4
FTO/perovskite/**V1387**	59.1	82.8	40.9	287.6	227.5

a The average decay time is calculated according to the equation τ_ave_ = (*A*_1_*τ*_1_^2^ + *A*_2_τ_2_^2^)/(*A*_1_τ_1_ + *A*_2_τ_2_).

**Figure 6:**
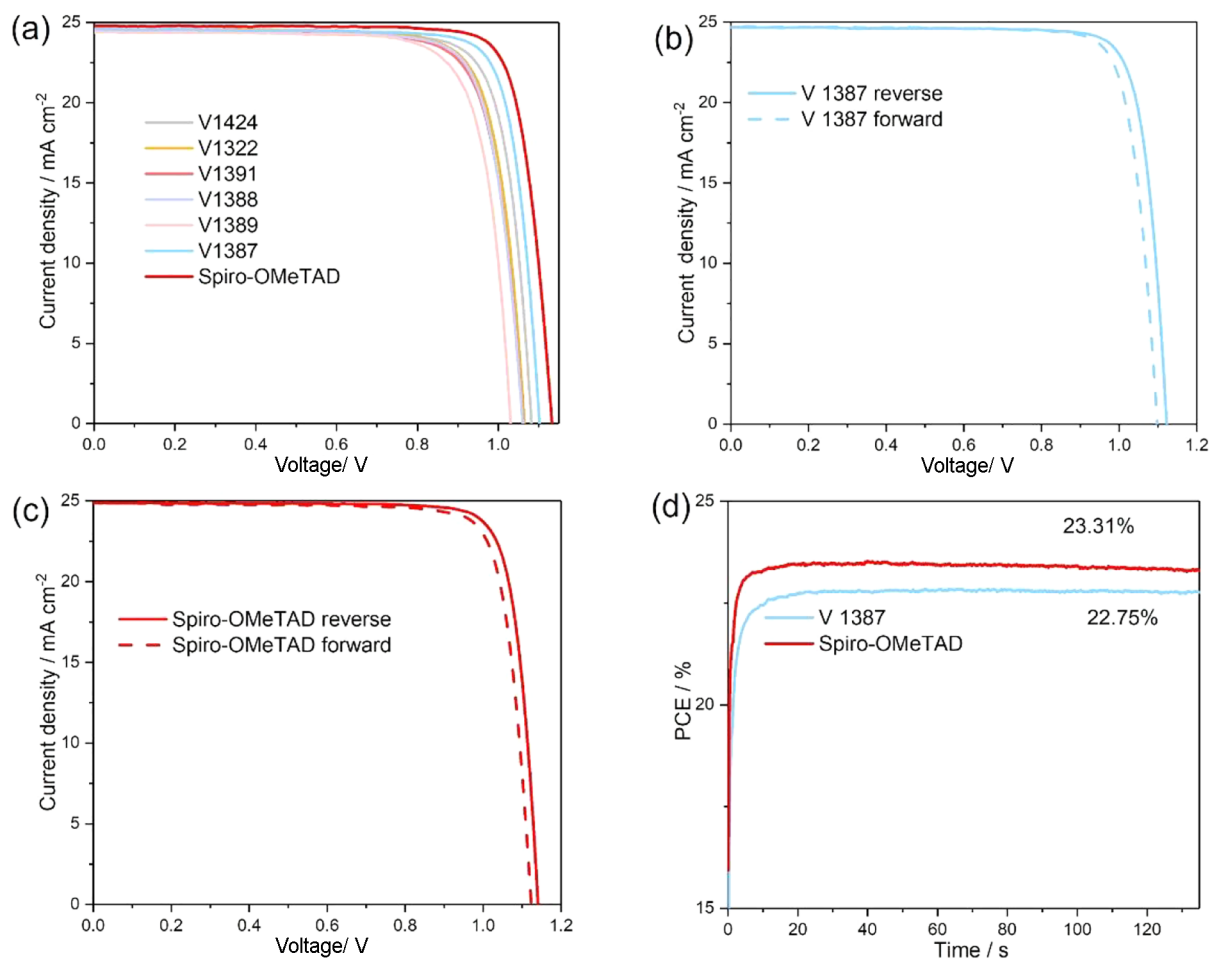
(a) *J–V* curves of the PSCs based on the various HTMs. (b) Reverse and forward *J–V* curves of the PSCs based on **V1387**. (c) Reverse and forward *J–V* curves of the PSCs based on spiro-OMeTAD. (d) The steady state efficiencies of the champion PSCs based on spiro-OMeTAD (red) and **V1387** (blue).

Next, the solar cells were characterized under simulated solar illumination by measuring the current density as a function of the applied voltage (*J–V* curves; see Figure 6a). The characteristic photovoltaic parameters of the PSCs with different HTMs were extracted from the *J–V* scans and are reported in Table 3, where the results of the devices are arranged in order from lowest to highest PCE. All of the HTMs were doped with lithium bis(trifluoromethanesulfonyl)imide (LiTFSI) in order to improve hole mobility. An analysis of the results suggests that the efficiency of the PSC decreases as the central core size of the HTM increases, since the PSCs with the HTMs that had bulkier TPA and carbazole core units (**V1389** and **V1391**, respectively) demonstrated the lowest PCE values. Conversely, out of all the new HTMs tested in PSCs, the best results were achieved with compounds that possessed the highest HOMO levels, **V1387** and **V1424**, which differ from one another only in the positions of the substitutions on the phenyl ring. However, this small structural variation between **V1387** and **V1424** results in an almost 1% difference in PCE, with **V1387** reaching a significantly high efficiency of 22.13%. In comparison, the benchmark HTM spiro-MeOTAD slightly outperformed the **V1387** devices (22.83%), delivering a PCE of 23.42% (Table S1, Figures 6b,c and S3). The *J–V* hysteresis of the champion PSCs can be observed in Figures 6b,c and S3. Low hysteresis indexes of 1.04 for **V1387**-based solar cells and 1.03 for spiro-OMeTAD-based solar cells were determined, which demonstrate the high quality of the fabricated PSCs. The steady state efficiencies were measured under AM1.5G illumination and are shown in Figure 6d. The devices based on spiro-OMeTAD and on **V1387** exhibit stable performances after 120 s output and show PCE values of 23.31% and 22.75%, respectively. It is clear that these values are extremely close to those obtained from the *J*–*V* curves. In addition, the incident photon to current efficiency (IPCE) of the devices based on **V1387** was determined to be 24.17 mA cm^–2^ (Figure S4), which is consistent with the short-circuit current (*J*_sc_) values.

**Table 3:** Photovoltaic Parameters Extracted from PSCs Based on the Various HTMs

HTMs	*J*_sc_ (mA cm^–2^)	*V*_oc_ (V)	fill factor, FF	PCE (%)
**V1389**	24.38	1.03	0.781	19.61
**V1391**	24.45	1.061	0.788	20.44
**V1388**	24.43	1.06	0.796	20.61
**V1322**	24.54	1.065	0.793	20.72
**V1424**	24.50	1.082	0.802	21.26
**V1387**	24.57	1.112	0.810	22.13
spiro-OMeTAD	24.79	1.133	0.813	22.83

**Figure 7:**
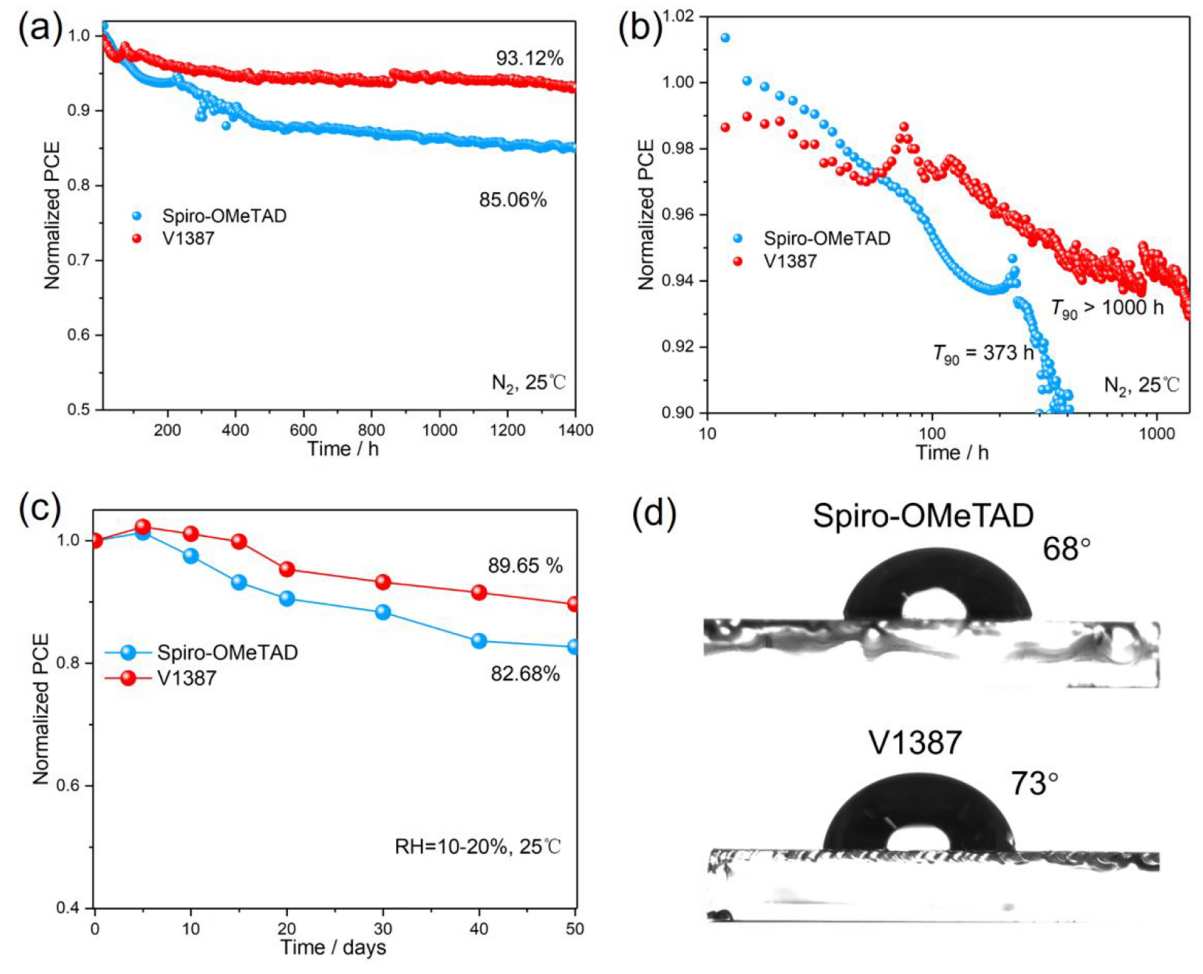
(a) MPP stability of the PSCs based on spiro-OMeTAD and **V1387** under the N_2_ storage condition (25 °C). (b) *T*_90_ stability of the PSCs based on spiro-OMeTAD and **V1387**. (c) Ambient stability of the PSCs based on spiro-OMeTAD and **V1387**. (d) The contact angles of the spiro-OMeTAD and **V1387** films.

Maximum power point (MPP) tracking of the devices employing **V1387** and spiro-OMeTAD was compared under N_2_ conditions (Figure 7a). Remarkably, the device with **V1387** retained 93.12% of its initial efficiency after a 1400 h output, whereas the device with spiro-OMeTAD maintained only 85.06% of the initial PCE under the same conditions. Figure 7b shows the *T*_90_ values of the stability of the devices. It can be seen that the device based on spiro-OMeTAD is less stable and reaches the *T*_90_ line quickly, in only 373 h. In addition, the **V1387**-based PSCs exhibit better ambient stability than devices based on spiro-OMeTAD (Figure 7c). Water contact angle (WCA) measurements reveal that the **V1387** film doped with LiTFSI exhibits a higher WCA (73°) than the spiro-OMeTAD-based films under the same conditions (68°). Hence, the greater hydrophobicity of **V1387** partially explains the high stability of the device that contains this HTM.

## 3 Conclusions

In this study, the synthesis and application of new HTMs that are composed of various central core units and substituted carbazole derivatives were developed in a facile two-step synthesis procedure. Their thermal, optical, and photoelectrical properties, as well as the PSC devices and interfacial transportation performances, were thoroughly investigated. All of the novel tested HTMs were successfully applied in PSCs, reaching an efficiency of up to 22.83%. Furthermore, the device employing the best performing HTM, **V1387**, demonstrated improved long-term stability compared to PSCs that used spiro-MeOTAD as a *p*-type organic charge carrier.

## 4 Experimental Section

### 4.1 Materials

Titanium diisopropoxide bis(acetylacetonate) (TAA), 4-*tert*-butylpyridine (*t*-BP), tin(IV) chloride pentahydrate (SnCl_4_), bis(trifluoromethane) sulfonamide lithium salt (LiTFSI), FK209 [tris(2-(1*H*-pyrazol-1-yl)-4-*tert*-butylpyridine)-cobalt(III)-tris(bis(trifluoromethylsulfonyl)imide)], chlorobenzene, dimethyl sulfoxide (DMSO), and dimethylformamide (DMF) were supplied from Sigma-Aldrich. Mesoporous TiO_2_ (30-NRT), FAI, MAI, and MACl were purchased from GreatCell Solar. HAT6 and PbI_2_ were purchased from TCI. Spiro-OMeTAD was purchased from Merck. All of the purchased chemicals were used as received without further purification.

### 4.2 Device Fabrication

For each device that was created, chemically etched FTO glass (Nippon Sheet Glass) was first cleaned with a detergent solution, deionized water, acetone, and isopropanol. To create the compact TiO_2_ (c-TiO_2_) layer, a TAA solution in ethanol (1.2 mL of TAA in 20 mL of anhydrous isopropanol) was sprayed at 450 °C and was further heated for 1 h at 450 °C. Then, mesoporous TiO_2_ (m-TiO_2_) paste was diluted with ethanol with a ratio of 1:10, coated on top of the c-TiO_2_ substrate at a speed of 3000 rpm for 20 s, and finally heated at 500 °C for 20 min. The tin oxide layer was formed by dissolving SnCl_4_ in deionized water at a concentration of 12 μL/mL, spin-coating that solution on the mesoporous TiO_2_ layer at a speed of 3000 rpm for 20 s, and heating the substrate at 190 °C for 60 min. Next, perovskite precursor solutions in DMSO/DMF 1:4 v/v (CsI = 11.78 mg; MAI = 11.12 mg; FAI = 228.72 mg; PbI_2_ = 709.95 mg; MACl = 18.90 mg) were successively spin-coated onto the substrate at 1000 rpm for 10 s and 5000 rpm for 30 s, consecutively. Then, 200 μL of chlorobenzene was dropped on the substrate for 10 s at 5000 rpm, and the perovskite films were annealed at 150 °C for 10 min. The spiro-OMeTAD HTM solution was prepared by dissolving 80 mg of spiro-OMeTAD (Merck) in 1 mL of chlorobenzene. The novel HTMs of this study were prepared by dissolving 50 mg of each compound in 1 mL of chlorobenzene. The following additives were added: 18 μL of LiTFSI from the stock solution (520 mg in 1 mL of acetonitrile), 13 μL of FK209 [tris(2-(1*H*-pyrazol-1-yl)-4-*tert*-butylpyridine)-cobalt(III)-tris(bis(trifluoromethylsulfonyl)imide)] (375 mg in 1 mL of acetonitrile), and 30 μL of 4-*tert*-butylpyridine. The HTM layer was formed by spin-coating the desired HTM solution at 4000 rpm for 20 s in order to achieve a thickness of 70 nm. Finally, deposition of the Au electrode completed the device. All of the preparative work done to deposit the perovskite and spiro-OMeTAD was carried out in a N_2_-filled glovebox.

### 4.3 Device Characterization

The samples were prepared by spin-coating the HTM solution in chlorobenzene onto a FTO film (2000 rpm, 20 s); they were then irradiated by a 450 W Xe lamp filtered through a double monochromator (5 nm bandpass). The film morphology was investigated by using a high-resolution scanning electron microscope (SEM) (Merlin, Zeiss) that was equipped with a GEMINI II column and a Schottky field emission gun. Images were acquired with an in-lens secondary electron detector. For the PL lifetime measurements, samples were excited with a 408 nm pulsed laser (MDL 300, PicoQuant) with a 40 μm cm^2^ pulse energy density. Current–voltage characteristics were recorded by applying an external potential bias to the cell while recording the generated photocurrent with a digital source meter (Keithley Model 2400). The light source was a 450 W Xe lamp (Oriel), equipped with a Schott K113 Tempax sunlight filter (Präzisions Glas & Optik GmbH) in order to match the emission spectrum of the lamp to the AM1.5G standard. Before each measurement, the exact light intensity was determined by using a calibrated Si reference diode that was equipped with an infrared cutoff filter (KG3, Schott). The cells were masked with an active area of 0.09 cm^2^. Contact angle measurements were done with the help of a DSA30 drop shape analyzer instrument and analyzed with the help of the Krüss ADVANCE software.

### 4.4 Ionization Potential Measurements

The ionization potential was investigated by using the electron photoemission method, and the study was performed in air. The sample solutions (THF) were poured onto an aluminum-coated polyester film that was coated with an adhesive layer of a methyl methacrylate and methacrylic acid copolymer. A diffraction grating monochromator with a deuterium lamp was used for the experiment. The power of the falling light was ∼5 × 10^–8^ W. A negative voltage (−100 V) was connected to the test sample. The electron photoemission current was measured with an open Geiger–Müller counter. The measurement method error was evaluated to be 0.03 eV.

### 4.5 Hole Drift Mobility Measurements

Carrier drift mobility was determined by the time-of-flight (XTOF) method. The material solution (THF) was poured onto aluminum-coated glass plates. The sample was poured from a solution of a pure substance, and the layers were dried for 1 h at 60 °C. The thickness of the transport layer was measured with an optical microscope-interferometer. The drift mobility of the holes (μ) was determined by using the electrophotographic mode with an electric field of (0.1/1) × 10^6^ V/cm. Charge carriers were generated at the layer surface by illumination with a nitrogen laser using nanosecond pulses (λ = 337 nm). In most cases, the layers of pure material produced for the hole transport studies were of poor quality due to cracking and were not suitable for XTOF measurements due to rapid discharging. Therefore, charge transfer in layers of blends with bisphenol Z-polycarbonate (PC-Z), in weight ratios of 1:1, was used.

### 4.6 Study of Quantum Chemistry

Simulations of the ground state molecular structures for several of the most probable conformers were provided using Gaussian 16 software by means of density functional theory (DFT) via the B3LYP method and a 6-31G(d) basis set, supplemented with polarization functions (d). A list of several of the most probable molecular conformations is presented in Table S2. Two projections (*xy* and *xz*) of the mentioned conformations are presented in Figures S5–S10.

Electronic excitations were simulated using the semi-empirical TD method (for singlets). The parameters of the transition between MOs, which are related to the population of “spectroscopic” states, are presented in Table S3. Spatial distributions of the electron density for the HOMO–1, HOMO, LUMO, and LUMO+1 are presented in Tables S4–S6.

## Supporting Information Available

Detailed synthetic procedures, DSC and SEM data, detailed photovoltaic parameters, *J–V* curves, and results of the performed quantum chemistry studies (PDF)
