# Supplementary material for: Branched Fluorenylidene Derivatives with Low Ionization Potentials as Hole-Transporting Materials for Perovskite Solar Cells
Source: Chem Mater. 2023 Jul 29;35(15):5914–23. doi: 10.1021/acs.chemmater.3c00708 (PMC10413965; doi:10.1021/acs.chemmater.3c00708)
Supplement: Supplementary file 1 — cm3c00708_si_001.pdf [file cm3c00708_si_001.pdf]

Branched fluorenylidene derivatives with low ionization potentials as hole-transporting materials for perovskite solar cells

Supporting Information

Aistė Jegorovė,[a†] Jianxing Xia,[b†] Matas Steponaitis,[a] Maryte Daskeviciene,[a] Vygintas Jankauskas,[c] Alytis Gruodis,[c] Egidijus Kamarauskas,[c] Tadas Malinauskas,[a] Kasparas Rakstys,[a] Khalid A Alamry,[d] Vytautas Getautis[a] and Mohammad Khaja Nazeeruddin[b]**

[a] Department of Organic Chemistry, Kaunas University of Technology, Radvilenu pl. 19, Kaunas, 50254 Lithuania

[b] Institute of Chemical Sciences and Engineering, École Polytechnique Federale de Lausanne (EPFL), Lausanne, 1015 Switzerland

[c] Institute of Chemical Physics Vilnius University, Sauletekio al. 3, Vilnius 10257, Lithuania

[d] Chemistry Department, Faculty of Science, King Abdulaziz University, P. O. Box 80203, Jeddah, 21589, Saudi Arabia

† A. J. and X. J. contributed equally to this work.
*Corresponding authors:

vytautas.getautis@ktu.lt; mdkhaja.nazeeruddin@epfl.ch

General methods

Chemicals required for the synthesis were purchased from Sigma-Aldrich and TCI Europe and used as received without additional purification. New fluorene class compounds were synthesized following previously described procedures. ^{1}H NMR spectra were recorded at 400 MHz on a Bruker Avance III spectrometer, ^{13}C NMR spectra were collected using the same instrument at 101 MHz. The chemical shifts, expressed in ppm, were relative to tetramethylsilane (TMS). All the experiments were performed at 25 °C. Reactions were monitored by thin-layer chromatography on ALUGRAM SIL G/UV254 plates and developed with UV light. Silica gel (grade 9385, 230–400 mesh, 60 Å, Aldrich) was used for column chromatography.

UV/VIS spectral analysis of the sample solutions (THF, 10^{-4} mol/l) and thin films was performed on a Perkin Elmer Lambda 35 UV / VIS spectrophotometer. The layer thickness of the solution is d = 1 mm. Diffraction grating crack width 2 nm. Spectral recording speed 2 nm / s. The wavelength λ is given in nm. Elemental analysis was performed with an Exeter Analytical CE-440 elemental analyzer, Model 440 C/H/N/.

The transformations of thermal changes of target materials were recorded with a TA Instruments Q2000 differential scanning calorimeter in a nitrogen atmosphere. From the obtained thermal curves, the glass transition temperature of the materials is determined. Heating and cooling mode reaches 10 ° C / min. The destruction temperatures of the new compounds were recorded by TA Instruments Q50 by thermogravimetric analysis in a nitrogen atmosphere. The decomposition temperature of a compound is recorded when the weight loss is 5%. Heating mode 20 ° C / min.

Compounds **1-5** were synthesized following procedures reported in the literature.[1] 9-ethyl-*N*-(4-metoxyphenyl)-9*H*-carbazol-3-amine was synthesized by Buchwald-Hartwig amination using Pd catalyst. Spectral data is in agreement with the values reported in the literature [2].

Synthesis

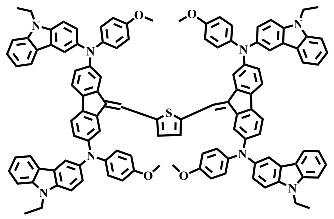

9,9'-(thiophene-2,5-diylbis(methanylylidene))bis(N^2,N^7-bis(9-ethyl-9*H*-carbazol-3-yl)-N^2,N^7-bis(4-methoxyphenyl)-9*H*-fluorene-2,7-diamine (V1322)

Reaction mixture of 2,5-bis((2,7-dibromo-9*H*-fluoren-9-ylidene)methyl)thiophene (**1**) (0.45 g, 0.6 mmol), 9-ethyl-N-(4-methoxyphenyl)-9*H*-carbazol-3-amine (1.13 g, 3.6 mmol) and anhydrous toluene (8 mL) was purged with Argon for 30 minutes. Afterwards, palladium (II) acetate (0.008 g, 0.03 mmol) and tri-*tert*-butylphosphonium tetrafluoroborate (0.02 g, 0.06 mmol) was added and stirred for 10 minutes under Argon. After that, sodium *tert*-butoxide (0.35 g, 3.6 mmol) was added and the mixture was heated at reflux for 5 hours (TLC control, THF/hexane 1:1.5). After cooling to room temperature, the reaction mixture was filtered through celite and extracted with ethyl acetate. The organic layer was dried by Na_2SO_4, filtered and the solvent was removed by vacuum rotary evaporation. The product was purified by column chromatography (eluent: THF/*n*-hexane 8:17 v:v). The final product was dissolved in THF and precipitated from ethanol to collect the final product as a brown solid powder. Yield 0.65 g, 64 %.

^{1}H NMR (400 MHz, THF-d_8) δ: 8.01 – 7.88 (m, 6H), 7.84 (s, 2H), 7.64 (s, 2H), 7.49 – 6.90 (m, 38H), 6.89 – 6.74 (m, 6H), 6.73 – 6.63 (m, 4H), 6.08 (s, 2H), 4.34 (q, *J* = 7.14 Hz, 4H), 4.16 (q, *J* = 7.14 Hz, 4H), 3.71 (s, 6H), 3.55 (s, 6H), 1.35 (t, *J* = 7.14 Hz, 6H), 1.25 (t, *J* = 7.14 Hz, 6H).

^{13}C NMR (101 MHz, THF-d_8) δ: 156.8, 156.5, 149.3, 148.9, 143.4, 142.4, 141.9, 141.60, 141.56, 141.5, 141.4, 140.8, 138.0, 137.9, 137.8, 136.9, 134.7, 134.1, 131.3, 127.2, 126.6, 126.5, 126.1, 125.9, 125.4, 125.0, 124.9, 123.8, 123.7, 121.3, 121.23, 121.16, 119.9, 119.4, 119.03, 118.96, 118.3, 118.0, 115.5, 115.3, 110.3, 110.2, 109.4, 55.6, 55.5, 38.14, 38.05, 14.3, 14.2.

Anal. Calcd. For $C_{116}H_{92}N_8O_4S$: C 82.24; H 5.47; N 6.61; O 3.78; S 1.89; found: C 82.31; H 5.42; N 6.59.

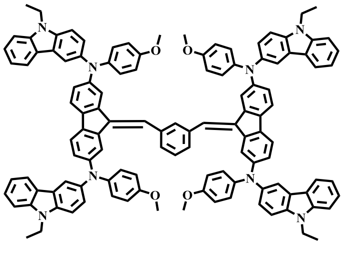

9,9'-(1,3-phenylenebis(methanylylidene))bis(N^2,N^7-bis(9-ethyl-9*H*-carbazol-3-yl)-N^2,N^7-bis(4-methoxyphenyl)-9*H*-fluorene-2,7-diamine (V1387)

Reaction mixture of 1,3-bis((2,7-dibromo-9*H*-fluoren-9-ylidene)methyl)benzene (**2**) (0.55 g, 0.7 mmol), 9-ethyl-*N*-(4-methoxyphenyl)-9*H*-carbazol-3-amine (1.33 g, 4.2 mmol) and anhydrous toluene (9.5 mL) was purged with Argon for 30 minutes. Afterwards, palladium (II) acetate (0.009 g, 0.04 mmol) and tri-*tert*-butylphosphonium tetrafluoroborate (0.023 g, 0.08 mmol) was added and stirred for 10 minutes under Argon. After that, sodium *tert*-butoxide (0.4 g, 4.2 mmol) was added and the mixture was heated at reflux for 8 hours (TLC control, THF/hexane 1:1.5). After cooling to room temperature, the reaction mixture was filtered through celite and extracted with ethyl acetate. The organic layer was dried by Na_2SO_4, filtered and the solvent was removed by vacuum rotary evaporation. The product was purified by column chromatography (eluent: THF/*n*-hexane 8:17 v:v). The final product was dissolved in toluene and precipitated from *n*-hexane to collect the final product as a light terracotta solid powder. Yield 0.7 g, 59 %.

^{1}H NMR (400 MHz, $CDCl_3$) δ: 7.96 (d, J = 7.8 Hz, 2H), 7.91 (s, 2H), 7.78 (d, J = 7.8 Hz, 2H), 7.64 (s, 2H), 7.46 – 7.29 (m, 14H), 7.29 – 7.24 (m, 4H); 7.21 – 6.94 (m, 16H), 6.94 – 6.70 (m, 14H), 6.56 (d, J = 8.7 Hz, 4H), 6.29 (d, J = 7.7 Hz, 2H), 4.30 – 4.11 (m, 8H), 3.71 (s, 6H), 3.62 (s, 6H), 1.43 – 1.26 (m, 12H).

^{13}C NMR (101 MHz, $CDCl_3$) δ: 155.3, 155.1, 147.8, 147.6, 142.3, 140.5, 140.3, 139.4, 137.4, 136.9, 136.8, 136.3, 135.7, 133.4, 126.9, 126.2, 125.7, 125.4, 125.2, 124.7, 123.8, 123.6, 122.7, 122.6, 122.3, 120.62, 120.55, 118.7, 118.4, 117.7, 115.5, 114.7, 114.4, 114.0, 109.2, 109.0, 108.5, 108.4, 55.5, 55.3, 37.6, 37.4, 13.93, 13.86.

Anal. Calcd. For $C_{118}H_{94}N_8O_4$: C 83.96; H 5.61; N 6.64; O 3.79; found: C 83.89; H 5.63; N 6.69.

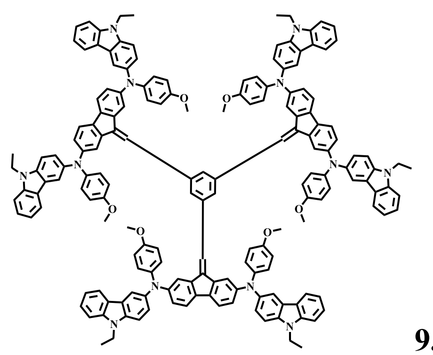

9,9',9''-(benzene-1,3,5-triyltris(methanylylidene))tris(N^2,N^7-bis(9-ethyl-9*H*-carbazol-3-yl)-N^2,N^7-bis(4-methoxyphenyl)-9*H*-fluorene-2,7-diamine (V1388)

Reaction mixture of 1,3,5-tris((2,7-dibromo-9*H*-fluoren-9-ylidene)methyl)benzene (**3**) (0.65 g, 0.6 mmol), 9-ethyl-*N*-(4-methoxyphenyl)-9*H*-carbazol-3-amine (1.7 g, 5.4 mmol) and anhydrous toluene (12 mL) was purged with Argon for 30 minutes. Afterwards, palladium (II) acetate (0.07 g, 0.03 mmol) and tri-*tert*-butylphosphonium tetrafluoroborate (0.020 g, 0.07 mmol) was added and stirred for 10 minutes under Argon. After that, sodium *tert*-butoxide (0.52 g, 5.4 mmol) was added and the mixture was heated at reflux for 8 hours (TLC control, THF/hexane 1:1.5). After cooling to room temperature, the reaction mixture was filtered through celite and extracted with ethyl acetate. The organic layer was dried by Na_2SO_4, filtered and the solvent was removed by vacuum rotary evaporation. The product was purified by column chromatography (eluent: THF/*n*-hexane 1:1.5 v:v). The final product was dissolved in toluene and precipitated from *n*-hexane to collect the final product as a light terracotta solid powder. Yield 0.57 g, 38 %.

^{1}H NMR (400 MHz, $CDCl_3$) δ: 8.00 – 7.89 (m, 6H), 7.59 (d, *J* = 7.7 Hz, 3H), 7.51 (s, 3H), 7.46 – 7.25 (m, 22H), 7.22 – 7.02 (m, 21H), 6.88 (t, *J* = 7.4 Hz, 3H), 6.85 – 6.76 (m, 8H), 6.74 – 7.62 (m, 12H), 6.50 (s, 3H), 6.43 (d, *J* = 8.6 Hz, 6H), 6.38 (s, 3H), 4.19 (q, *J* = 7.1 Hz, 6H), 3.94 (q, *J* = 7.1 Hz, 6H), 3.63 (s, 9H), 3.51 (s, 9H), 1.31 (t, *J* = 7.1 Hz, 9H), 1.18 (t, *J* = 7.1 Hz, 9H).

^{13}C NMR (101 MHz, $CDCl_3$) δ: 155.2, 155.1, 147.9, 147.3, 142.4, 141.4, 141.0, 140.4, 140.1, 139.4, 137.7, 136.8, 136.7, 136.3, 135.6, 133.0, 129.1, 128.3, 128.0, 126.6, 125.9, 125.8, 125.5, 125.3, 125.1, 124.8, 123.8, 123.3, 122.61, 122.57, 122.0, 120.6, 120.5, 119.0, 118.7, 118.6, 117.8, 114.8, 114.3, 114.1, 109.3, 109.0, 108.6, 108.3, 55.5, 55.2, 37.5, 37.3, 13.9, 13.8.

Anal. Calcd. For $C_{174}H_{138}N_{12}O_6$: C 83.83; H 5.58; N 6.74; O 3.85; found: C 83.87; H 5.55; N 6.73.

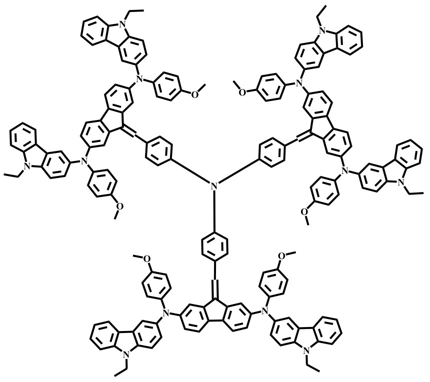

9-(4-bis(4-((2,7-bis((9-9-ethyl-9*H*-carbazol-3-yl)(4-methoxyphenyl)amino)-9*H*-fluoren-9-ylidene)methyl)phenyl)amino)benzylidene-*N*^{2},*N*^{7}-bis(4-methoxyphenyl)-9*H*-fluorene-2,7-diamine (V1389)

Reaction mixture of tris(4-((2,7-dibromo-9*H*-fluoren-9-ylidene)phenyl)amine (**4**) (0.6 g, 0.5 mmol), 9-ethyl-*N*-(4-methoxyphenyl)-9*H*-carbazol-3-amine (1.42 g, 4.5 mmol) and anhydrous toluene (10 mL) was purged with Argon for 30 minutes. Afterwards, palladium (II) acetate (0.07 g, 0.03 mmol) and tri-*tert*-butylphosphonium tetrafluoroborate (0.017 g, 0.06 mmol) was added and

stirred for 10 minutes under Argon. After that, sodium *tert*-butoxide (0.43 g, 4.5 mmol) was added and the mixture was heated at reflux for 5 hours (TLC control, THF/hexane 1.5:1). After cooling to room temperature, the reaction mixture was filtered through celite and extracted with ethyl acetate. The organic layer was dried by Na_2SO_4, filtered and the solvent was removed by vacuum rotary evaporation. The product was purified by column chromatography (eluent: THF/*n*-hexane 11:14 v:v). The final product was dissolved in toluene and precipitated from *n*-hexane to collect the final product as a brown solid powder. Yield 0.7 g, 53 %.

^{1}H NMR (400 MHz, $CDCl_3$) δ: 8.00 – 7.88 (m, 6H), 7.84 – 7.62 (m, 9H), 7.50 – 7.31 (m, 20H), 7.26 – 6.94 (m, 46H), 6.87 (d, *J* = 8.5 Hz, 6H), 6.67 (d, *J* = 8.4 Hz, 6H), 6.13 (d, *J* = 8.2 Hz, 6H), 4.35 (q, *J* = 7.2 Hz, 6H), 4.02 (q, *J* = 7.2 Hz, 6H), 3.81 (s, 9H), 3.51 (s, 9H), 1.45 (t, *J* = 7.2 Hz, 9H), 1.20 (t, *J* = 7.2 Hz, 9H).

^{13}C NMR (101 MHz, $CDCl_3$) δ: 145.8, 141.1, 140.4, 140.3, 130.5, 130.4, 125.8, 125.6, 123.3, 122.6, 122.4, 120.6, 120.4, 118.7, 118.6, 113.7, 109.2, 108.6, 55.5, 55.3, 37.7, 37.4, 14.0, 13.8.

Anal. Calcd. For $C_{186}H_{147}N_{13}O_6$: C 83.98; H 5.57; N 6.84; O 3.61; found: C 83.96; H 5.59; N 6.84.

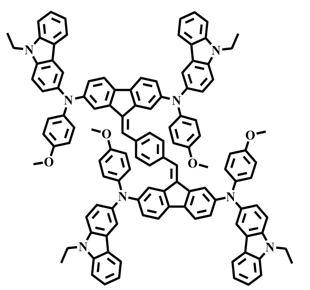

9,9'-(1,4-phenylenebis(methanylylidene))bis(N^2,N^7-bis(9-ethyl-9*H*-carbazol-3-yl)-N^2,N^7-bis(4-methoxyphenyl)-9*H*-fluorene-2,7-diamine (V1424)

Reaction mixture of 1,4-bis((2,7-dibromo-9*H*-fluoren-9-ylidene)methyl)benzene (**5**) (0.75 g, 1 mmol), 9-ethyl-*N*-(4-methoxyphenyl)-9*H*-carbazol-3-amine (1.9 g, 6 mmol) and anhydrous

toluene (13 mL) was purged with Argon for 30 minutes. Afterwards, palladium (II) acetate (0.013 g, 0.06 mmol) and tri-*tert*-butylphosphonium tetrafluoroborate (0.032 g, 0.1 mmol) was added and stirred for 10 minutes under Argon. After that, sodium *tert*-butoxide (0.58 g, 6 mmol) was added and the mixture was heated at reflux for 5 hours (TLC control, THF/hexane 1:1.5). After cooling to room temperature, the reaction mixture was filtered through celite and extracted with ethyl acetate. The organic layer was dried by Na_2SO_4, filtered and the solvent was removed by vacuum rotary evaporation. The product was purified by column chromatography (eluent: THF/*n*-hexane 8:17 v:v). The final product was dissolved in THF and precipitated from ethanol to collect the final product as a light brown solid powder. Yield 1 g, 59 %.

^{1}H NMR (400 MHz, $CDCl_3$) δ: 8.02 – 7.86 (m, 4H), 7.85 – 7.60 (m, 4H), 7.52 – 7.11 (m, 25H), 7.11 – 6.80 (m, 19H), 6.74 (s, 2H), 6.61 (d, J = 8.1 Hz, 4H), 6.34 (s, 4H), 4.35 (q, J = 7.3 Hz, 4H), 3.93 (q, J = 7.3 Hz, 4H), 3.81 (s, 6H), 3.58 (s, 6H), 1.44 (t, J = 7.3 Hz, 6H), 1.21 (t, J = 7.3 Hz, 6H).

^{13}C NMR (101 MHz, $CDCl_3$) δ: 155.1, 148.1, 147.8, 140.5, 140.2, 135.1, 128.2, 125.8, 125.6, 122.6, 122.5, 120.6, 120.3, 118.7, 118.6, 109.3, 108.6, 55.6, 55.2, 37.7, 37.3, 14.0.

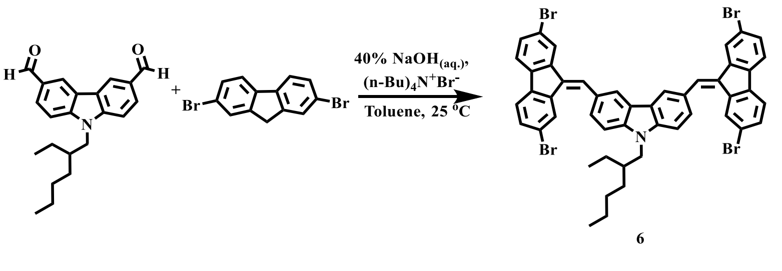

Scheme S1. Synthesis of fluorene derivative **6**.

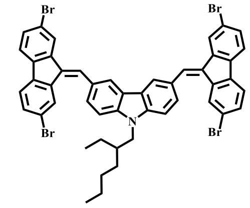

3,6-bis((2,7-dibromo-9*H*-fluoren-9-ylidene)methyl)-9-(2-ethylhexyl)-9*H*-carbazole (6)

A mixture of 2,7-dibromofluorene (1.86 g, 5.7 mmol), 9-(2-ethylhexyl)carbazole-3,6-dicarboxaldehyde (0.55 g, 1.6 mmol) and toluene (14.5 mL) was stirred for 10 minutes. Afterwards, NaOH solution (14.5 mL, 40% w/w) and tetrabutylammonium bromide (0.28 g, 0.88 mmol) were added and the reaction mixture was stirred at room temperature for 50 minutes (TLC control, acetone/*n*-hexane 1:4). After cooling to room temperature, the reaction mixture was extracted with ethyl acetate. The organic layer was dried over Na_2SO_4, filtered and the solvent was removed by vacuum rotary evaporation. The crude product was crystallized from THF/acetone mixture (1:1, v:v) to obtain orange crystals which were used without any further purification in the next step. Yield 0.8 g (52%).

<u>^{1}H NMR</u> (400 MHz, THF-d_8) δ 8.46 (s, 2H), 8.15 (d, J = 14.9 Hz, 4H), 8.05 (s, 2H), 7.84 (d, J = 8.5 Hz, 2H), 7.71 (d, J = 8.1 Hz, 6H), 7.48 (dd, J = 20.2, 8.1 Hz, 4H), 4.43 (d, J = 7.4 Hz, 2H), 1.60 – 1.39 (m, 6H), 1.37 – 1.28 (m, 2H), 1.00 (t, J = 7.4 Hz, 3H), 0.88 (m, 4H).

<u>^{13}C NMR</u> (101 MHz, THF) δ 140.05, 139.94, 137.12, 136.45, 135.48, 134.77, 130.72, 130.57, 129.10, 128.59, 126.02, 125.16, 124.70, 121.69, 121.30, 120.51, 120.01, 119.39, 119.17, 119.11, 118.50, 107.76, 65.14, 37.56, 29.04, 26.80, 23.01, 21.17, 11.58, 8.39.

Anal. Calcd. For $C_{118}H_{94}N_8O_4$: C 83.96; H 5.61; N 6.64; O 3.79; found: C 83.89; H 5.63; N 6.69.

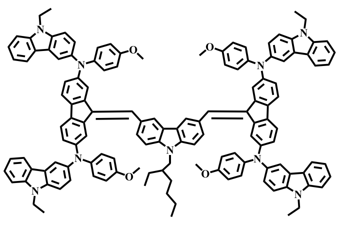

9,9'-((9-(2-ethylhexyl)-9*H*-carbazole-3,6-diyl)bis(methanylylidene))bis(*N*²,*N*⁷-bis(9-ethyl-9*H*-carbazol-3-yl)-*N*²,*N*⁷-bis(4-methoxyphenyl)-9*H*-fluorene-2,7-diamine (**V1391**)

Reaction mixture of 3,6-bis((2,7-dibromo-9*H*-fluoren-9-ylidene)methyl)-9-(2-ethylhexyl)- 9*H* -carbazole (**6**) (0.55 g, 0.6 mmol), 9-ethyl-*N*-(4-methoxyphenyl)-9*H*-carbazol-3-amine (1.14 g, 3.6 mmol) and anhydrous toluene (9 mL) was purged with Argon for 30 minutes. Afterwards, palladium (II) acetate (0.07 g, 0.03 mmol) and tri-*tert*-butylphosphonium tetrafluoroborate (0.02 g, 0.07 mmol) was added and stirred for 10 minutes under Argon. After that, sodium *tert*-butoxide (0.35 g, 3.6 mmol) was added and the mixture was heated at reflux for 5 hours (TLC control, THF/hexane 1:1.5). After cooling to room temperature, the reaction mixture was filtered through celite and extracted with ethyl acetate. The organic layer was dried by Na_2SO_4, filtered and the solvent was removed by vacuum rotary evaporation. The product was purified by column chromatography (eluent: THF/*n*-hexane 8:17 v:v). The final product was dissolved in THF and precipitated from ethanol to collect the final product as an orange solid powder. Yield 0.55 g, 49%.

^{1}H NMR (400 MHz, $CDCl_3$) δ: 7.96 (d, *J* = 7.8 Hz, 2H), 7.93 – 7.83 (m, 6H), 7.70 (d, *J* = 11.8 Hz, 4H), 7.56 – 7.49 (m, 4H), 7.48 – 7.26 (m, 20H), 7.19 – 6.97 (m, 12H), 6.92 (d, *J* = 8.4 Hz, 6H), 6.84 (d, *J* = 8.8 Hz, 4H), 6.63 (d, *J* = 8.8 Hz, 6H), 4.34 (q, *J* = 7.2 Hz, 4H), 4.11 (q, *J* = 7.2 Hz, 4H), 3.80 (s, 6H), 3.59 (s, 6H), 3.36 – 3.26 (m, 2H), 1.64 (s, 1H), 1.44 (t, *J* = 7.2 Hz, 6H), 1.27 (t, *J* = 7.2 Hz, 6H), 1.20 – 0.88 (m, 6H), 0.79 (t, *J* = 6.7 Hz, 3H), 0.69 (t, *J* = 6.7 Hz, 3H).

^{13}C NMR (101 MHz, $CDCl_3$) δ: 154.8, 141.3, 140.5, 140.5, 140.3, 137.7, 136.8, 136.4, 134.5, 128.8, 127.1, 126.9, 125.7, 125.6, 123.8, 123.5, 122.7, 122.6, 121.7, 120.7, 120.6, 119.3, 118.7,

118.6, 118.4, 114.6, 114.3, 113.9, 109.2, 108.9, 108.5, 77.4, 77.1, 76.7, 55.6, 55.4, 37.7, 37.5, 30.6, 28.5, 24.0, 23.0, 14.04, 13.98, 13.8, 10.8.

Anal. Calcd. For $C_{132}H_{113}N_9O_4$: C 83.91; H 6.03; N 6.67; O 3.39; found: C 83.87; H 6.06; N 6.68.

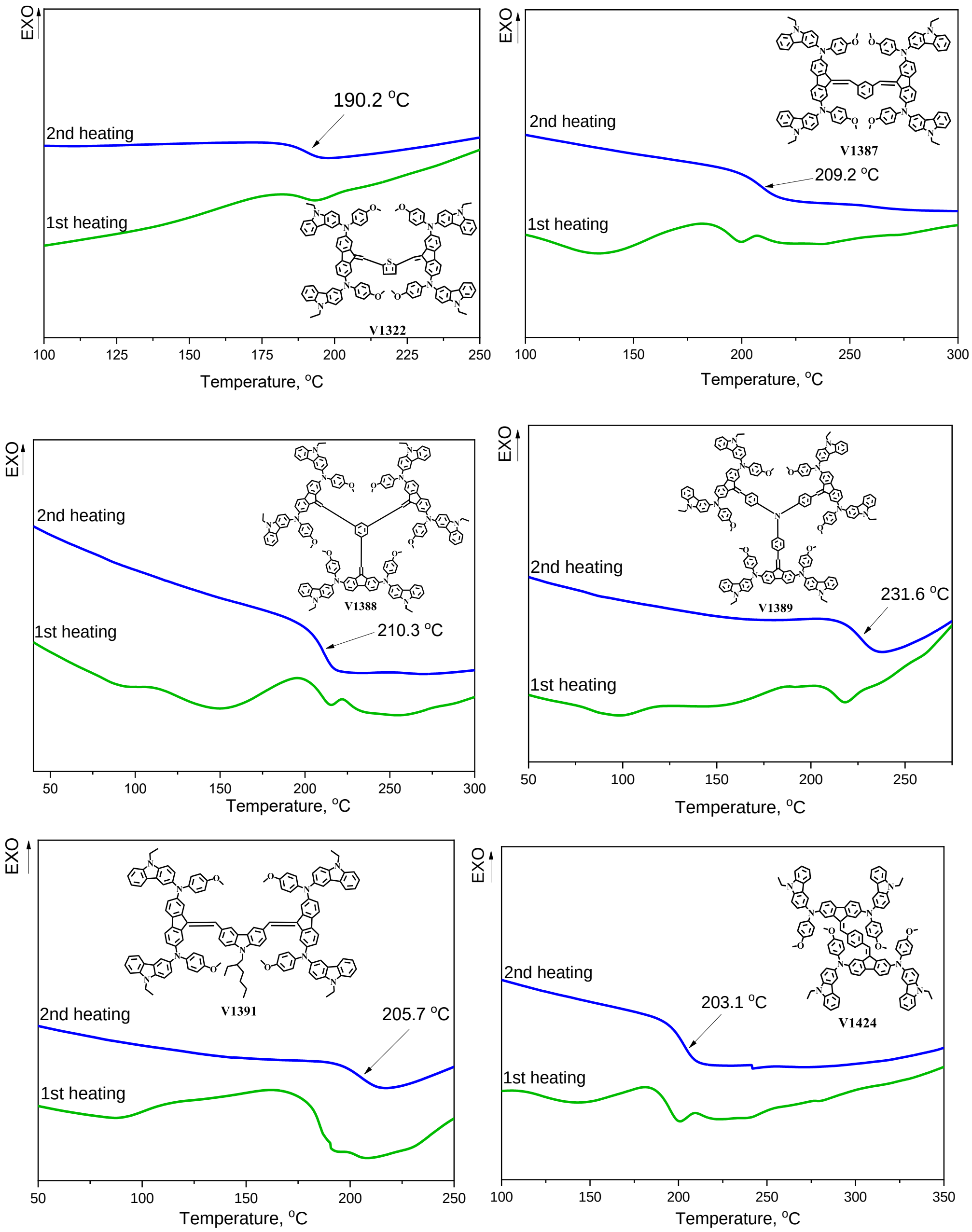

Figure S1. DSC curves of tested HTMs.

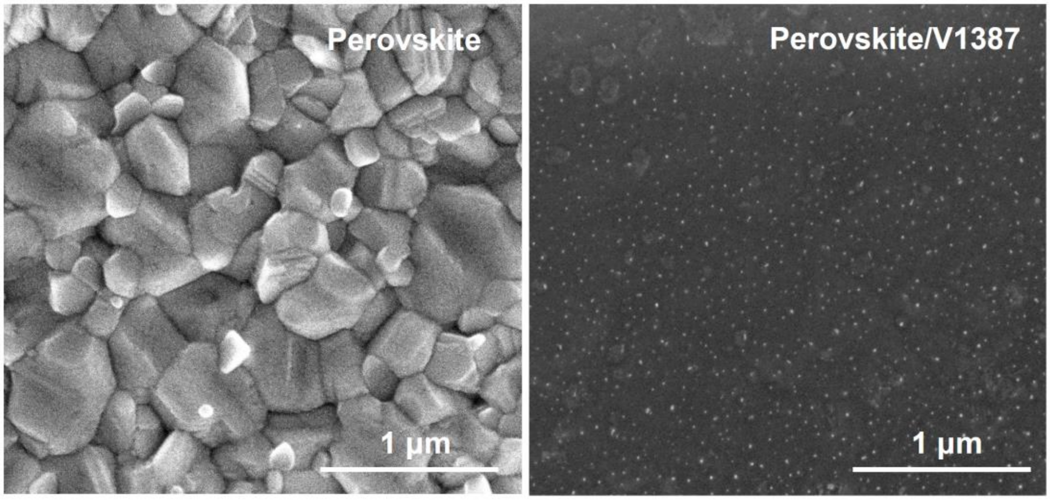

Figure S2. The surface SEM of perovskite and perovskite/**V1387** films.

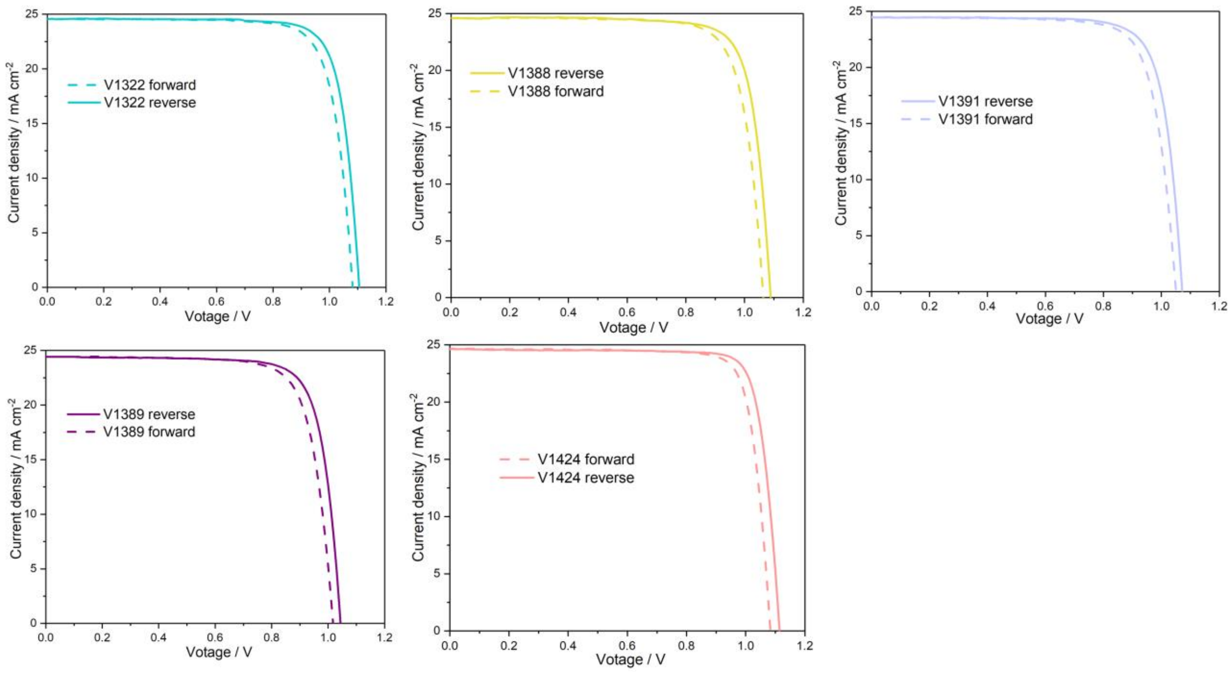

Figure S3. The *J-V* curves of optimized PSCs based on HTMs

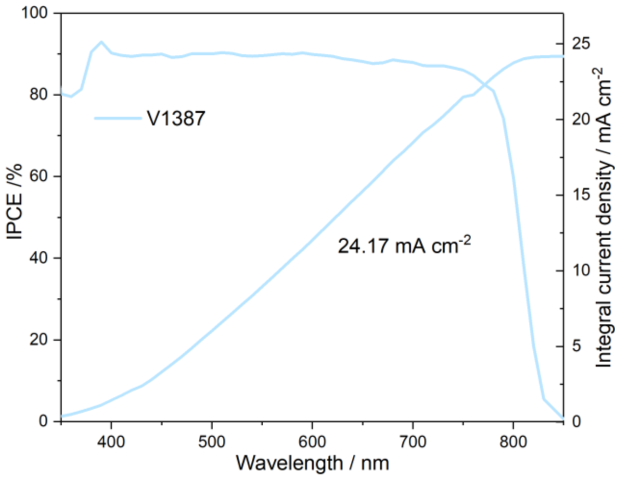

Figure S4. The IPCE of PSCs based on **V1387** HTM.

Table S1. Results of **V1387** and spiro-MeOTAD forward and reverse scans.

HTMs	J_{sc} [mA cm^{-2}]	V_{oc} [V]	FF	PCE [%]
**V1389 reverse**	24.41	1.043	0.791	20.13
**V1389 forward**	24.43	1.016	0.778	19.31
**V1391 reverse**	24.46	1.072	0.798	20.92
**V1391 forward**	24.49	1.05	0.784	20.16
**V1388 reverse**	24.60	1.09	0.805	21.58
**V1388 forward**	24.61	1.064	0.790	20.68
**V1322 reverse**	24.54	1.103	0.803	21.73
**V1322 forward**	24.56	1.082	0.795	21.12
**V1424 reverse**	24.63	1.115	0.820	22.51
**V1424 forward**	24.66	1.084	0.809	21.62
**V1387 reverse**	24.69	1.121	0.825	22.83
**V1387 forward**	24.72	1.097	0.810	21.96
**Spiro-OMeTAD reverse**	24.85	1.141	0.826	23.42
**Spiro-OMeTAD forward**	24.88	1.124	0.812	22.70

Table S2. List of molecular conformations of the compounds.

Compound	Two most probable conformers	Molecular structure after ground state energy optimization using *Gaussian16*, B3LYP/6-31G(d) basis set
**V1322**	V1322a, V1322b	Fig. S5
**V1387**	V1387a, V1387b	Fig. S6
**V1391**	V1391a, V1391b	Fig. S7
**V1424**	V1424a, V1424b	Fig. S8
**V1388**	V1388a, V1388b	Fig. S9
**V1389**	V1389a, V1389b	Fig. S10

xy

xz

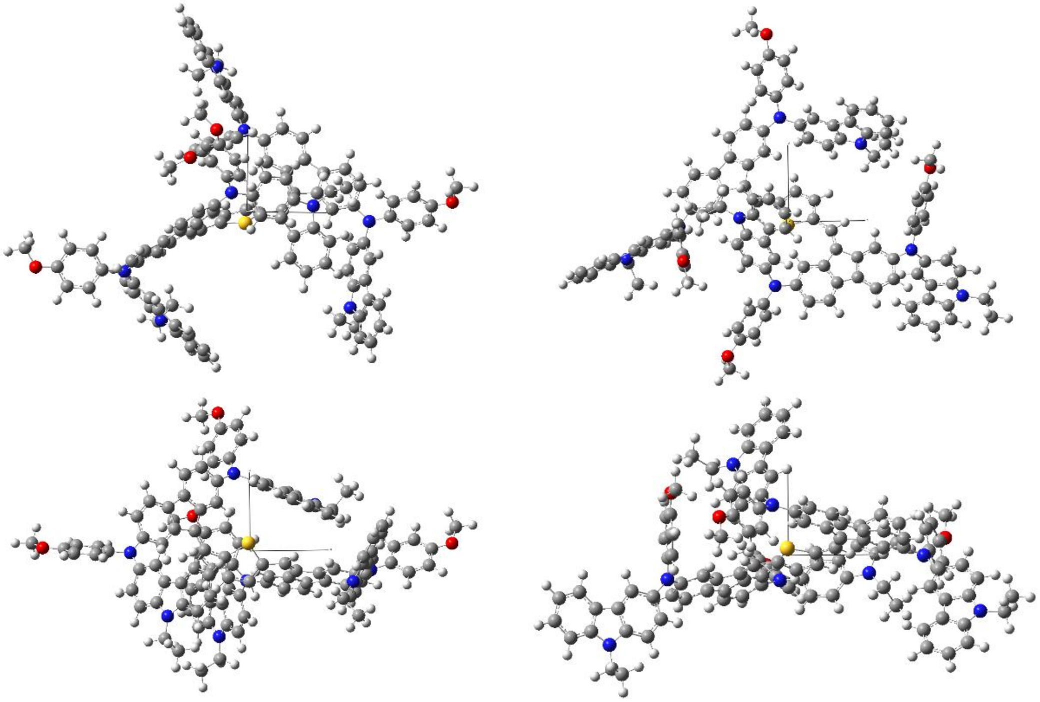

V1322a **V1322b**

Fig. S5. Molecular structure of two conformers **V1322** after ground state energy optimization routine using *Gaussian16*, B3LYP/6-31G(d) basis set. Two projections: xy and xz.

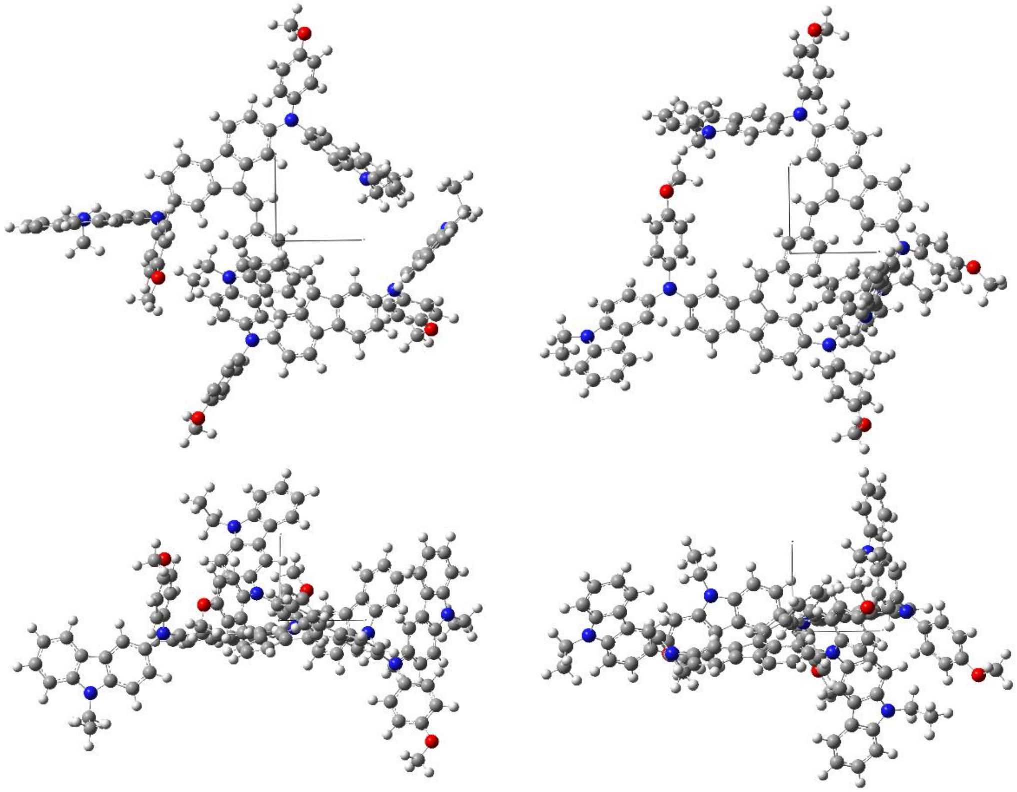

xy

xz

V1387a **V1387b**

Fig. S6. Molecular structure of two conformers **V1387** after ground state energy optimization routine using *Gaussian16*, B3LYP/6-31G(d) basis set. Two projections: xy and xz.

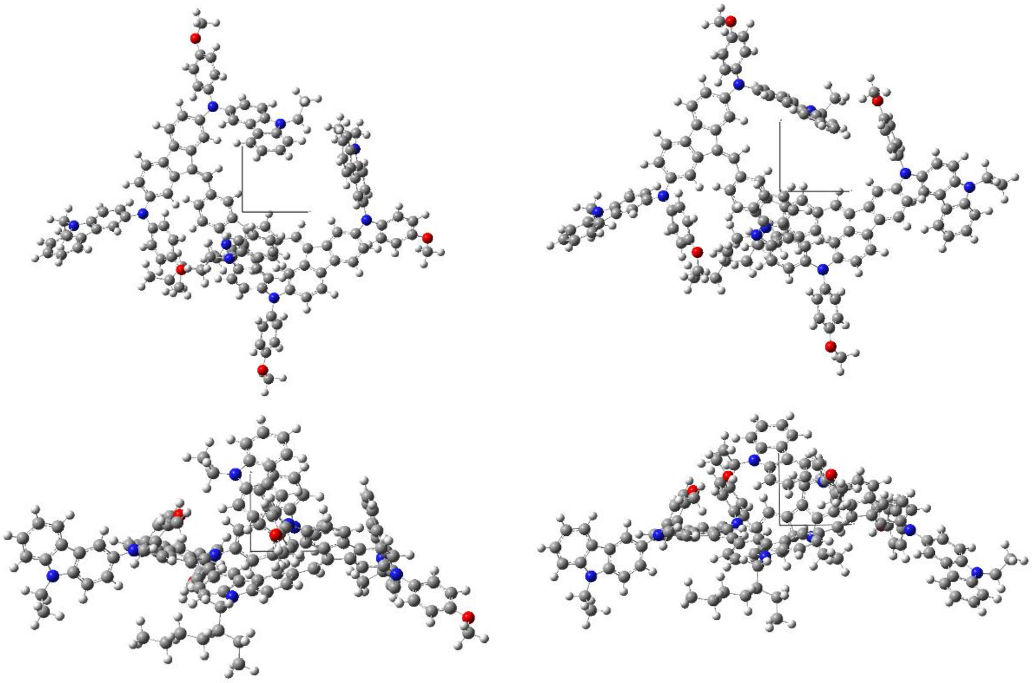

Fig. S7. Molecular structure of two conformers **V1391** after ground state energy optimization routine using *Gaussian16*, B3LYP/6-31G(d) basis set. Two projections: xy and xz.

xy

xz

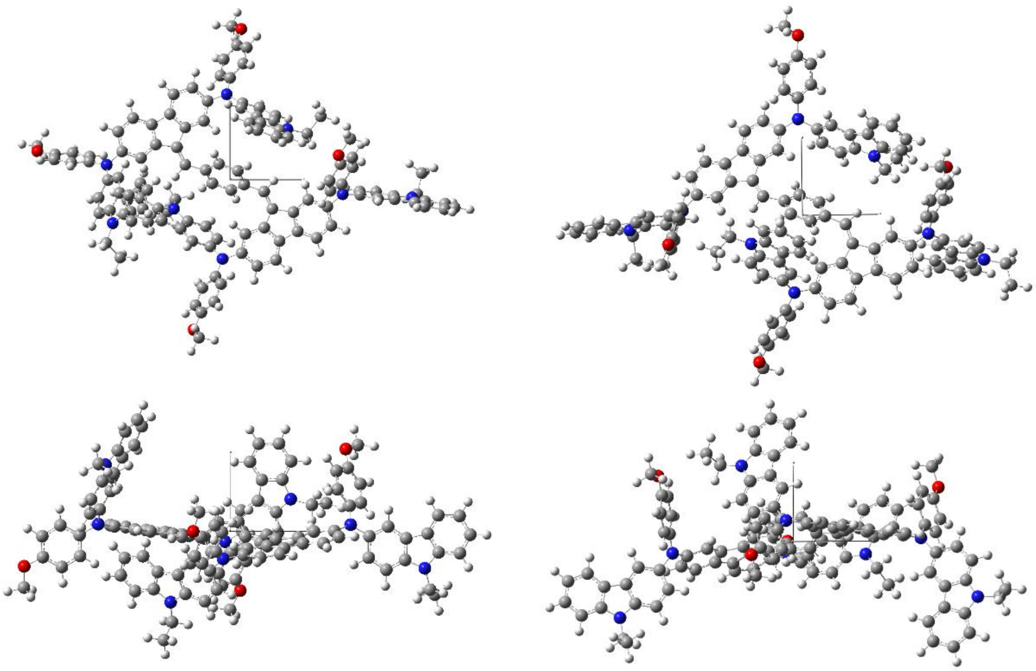

V1424a **V1424b**

Fig. S8. Molecular structure of two conformers **V1424** after ground state energy optimization routine using *Gaussian16*, B3LYP/6-31G(d) basis set. Two projections: xy and xz.

xy

xz

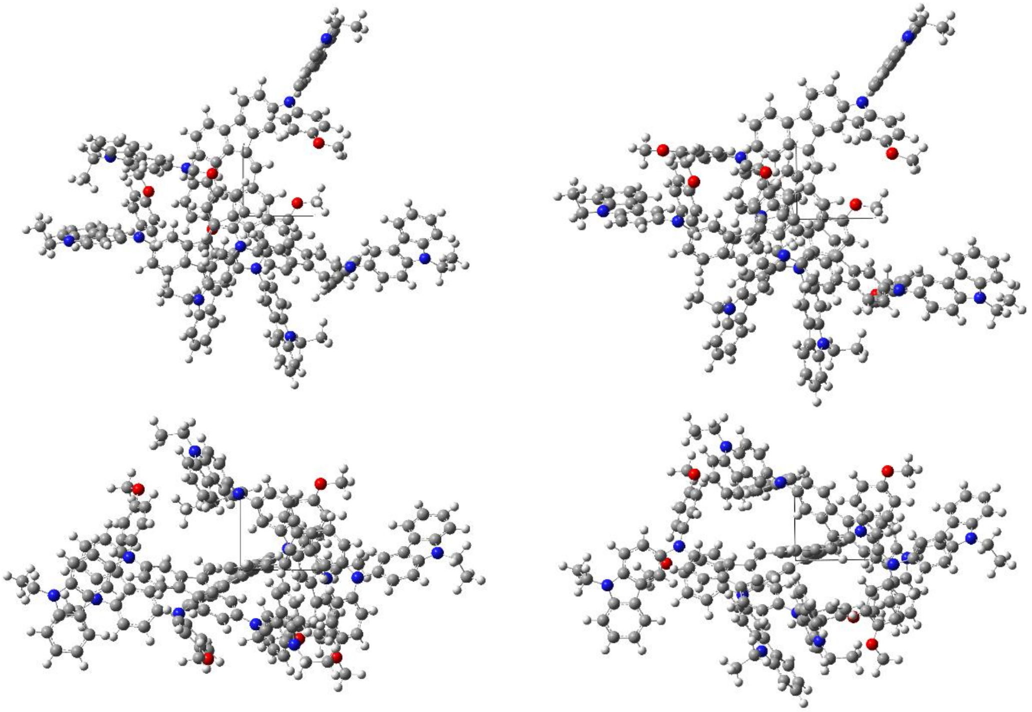

V1388a **V1388b**

Fig. S9. Molecular structure of two conformers **V1388** after ground state energy optimization routine using *Gaussian16*, B3LYP/6-31G(d) basis set. Two projections: xy and xz.

xy

xz

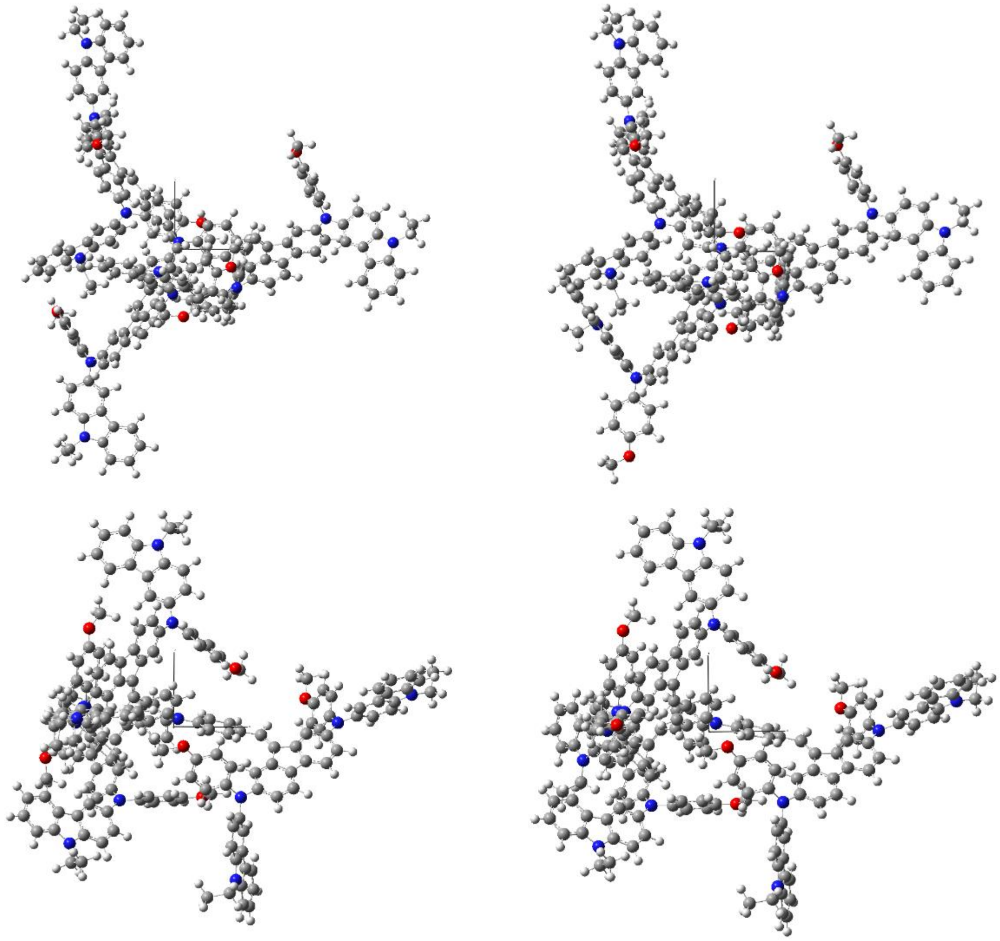

V1389a **V1389b**

Fig. S10. Molecular structure of two conformers **V1389** after ground state energy optimization routine using *Gaussian16*, B3LYP/6-31G(d) basis set. Two projections: xy and xz.

Table S3. Parameters of electronic excitations simulated using semiempirical TD method (for singlets). Population of "spectroscopic" states (from ground to excited) ΔE_n *and corresponding* set of MO with contribution coefficient *k* (contribution of the respective excitation to the configurational interaction wavefunction).

	a conformer			b conformer		
**V1322**	Δ*E*	Transition between MO	*k*	Δ*E*	Transition between MO	*k*
	$S_0 \rightarrow S_1$	HOMO→LUMO	0.70142	$S_0 \rightarrow S_1$	HOMO→LUMO	0.70237
	$S_0 \rightarrow S_2$	HOMO-1→LUMO	0.69954	$S_0 \rightarrow S_2$	HOMO-1→LUMO	0.70171
**V1387**	Δ*E*	Transition between MO	*k*	Δ*E*	Transition between MO	*k*
	$S_0 \rightarrow S_1$	HOMO→LUMO	0.65481	$S_0 \rightarrow S_1$	HOMO→LUMO	0.61076
	$S_0 \rightarrow S_2$	HOMO-1→LUMO	0.67076	$S_0 \rightarrow S_2$	HOMO-1→LUMO	0.61829
**V1391**	Δ*E*	Transition between MO	*k*	Δ*E*	Transition between MO	*k*
	$S_0 \rightarrow S_1$	HOMO→LUMO	0.60654	$S_0 \rightarrow S_1$	HOMO→LUMO	0.63617
	$S_0 \rightarrow S_2$	HOMO-1→LUMO	0.62024	$S_0 \rightarrow S_2$	HOMO-1→LUMO	0.60910
**V1424**	Δ*E*	Transition between MO	*k*	Δ*E*	Transition between MO	*k*
	$S_0 \rightarrow S_1$	HOMO→LUMO	0.69544	$S_0 \rightarrow S_1$	HOMO→LUMO	0.69727
	$S_0 \rightarrow S_2$	HOMO-1→LUMO	0.69880	$S_0 \rightarrow S_2$	HOMO-1→LUMO	0.69735
**V1388**	Δ*E*	Transition between MO	*k*	Δ*E*	Transition between MO	*k*
	$S_0 \rightarrow S_1$	HOMO-1→LUMO	0.66229	$S_0 \rightarrow S_1$	HOMO-2→LUMO	0.69602
	$S_0 \rightarrow S_2$	HOMO→LUMO	0.56232	$S_0 \rightarrow S_2$	HOMO→LUMO	0.61174
		HOMO→LUMO+1	0.34090			
**V1389**	Δ*E*	Transition between MO	*k*	Δ*E*	Transition between MO	*k*
	$S_0 \rightarrow S_1$	HOMO→LUMO	0.63076	$S_0 \rightarrow S_1$	HOMO→LUMO	0.65525
	$S_0 \rightarrow S_2$	HOMO-1→LUMO	0.55796	$S_0 \rightarrow S_2$	HOMO-1→LUMO	0.53053
					HOMO-1→LUMO+1	-0.40009

Table S4. Compounds **V1322** and **V1387**. Set of MO involved into "spectroscopic" states $S_0 \rightarrow S_1$ and $S_0 \rightarrow S_2$

MO	**V1322a**	**V1322b**	**V1387a**	**V1387b**
LUMO+1	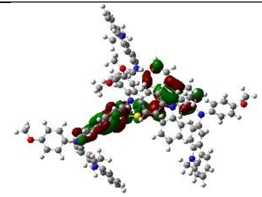	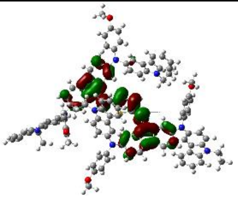	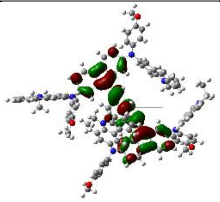	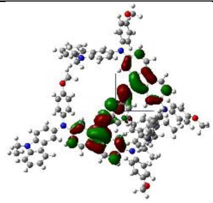
LUMO	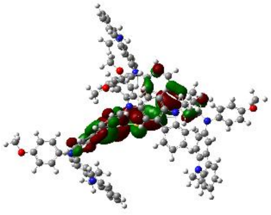	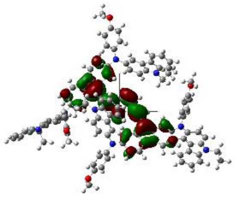	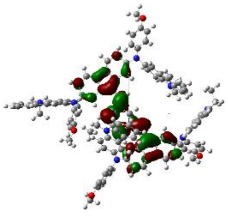	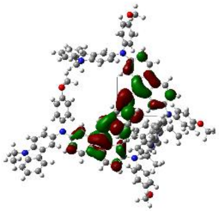
HOMO	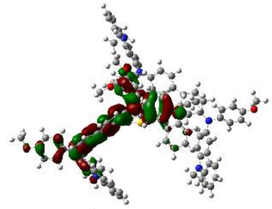	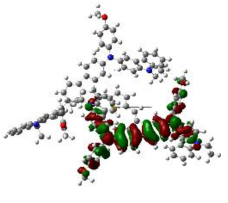	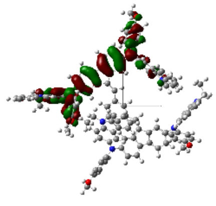	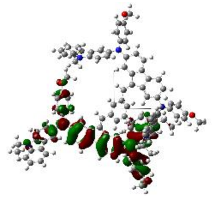
HOMO-1	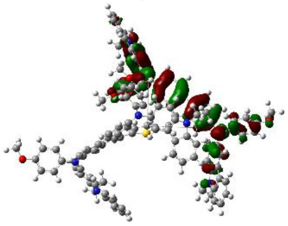	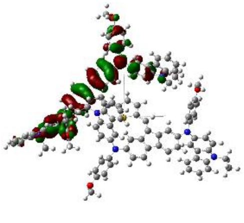	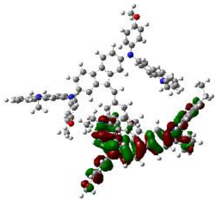	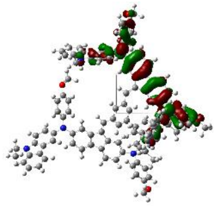

Table S5. Compounds **V1391** and **V1424**. Set of MO involved into “spectroscopic” states $S_0 \rightarrow S_1$ and $S_0 \rightarrow S_2$

MO	**V1391a**	**V1391b**	**V1424a**	**V1424b**
LUMO+1	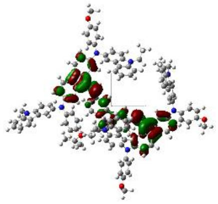	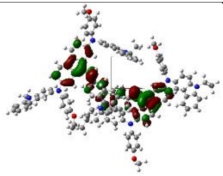	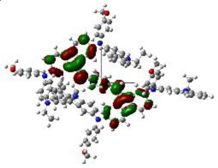	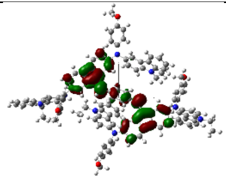
LUMO	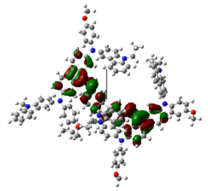	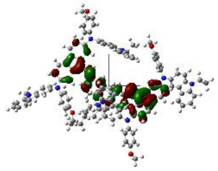	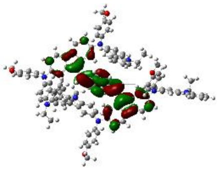	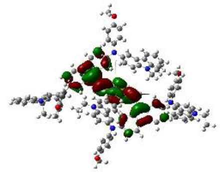
HOMO	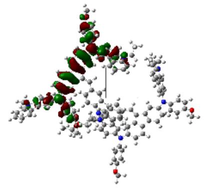	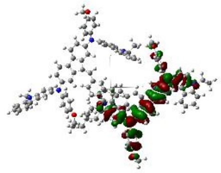	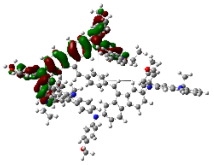	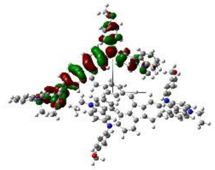
HOMO-1	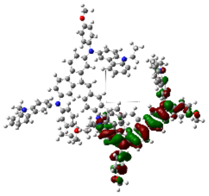	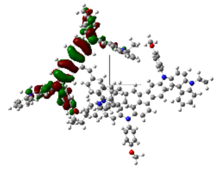	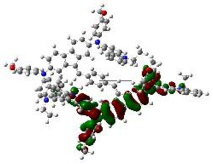	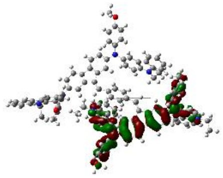

Table S6. Compounds **V1388** and **V1389**. Set of MO involved into "spectroscopic" states $S_0 \rightarrow S_1$ and $S_0 \rightarrow S_2$

MO	**V1388a**	**V1388b**	**V1389a**	**V1389b**
LUMO+1	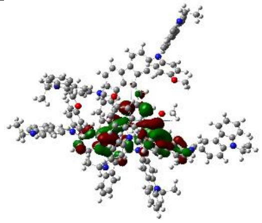	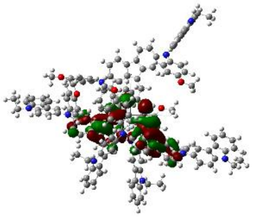	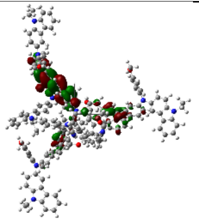	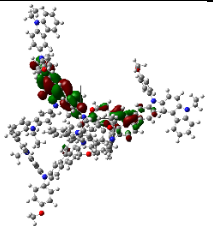
LUMO	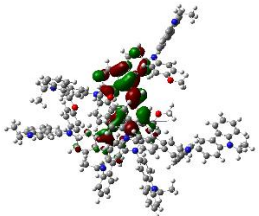	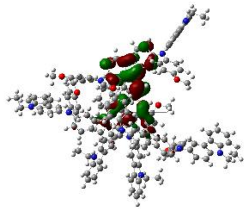	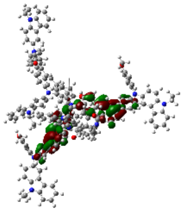	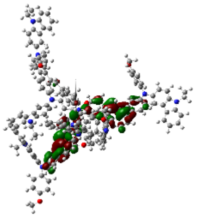
HOMO	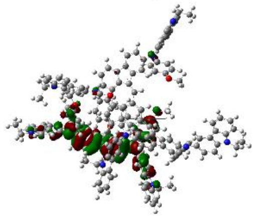	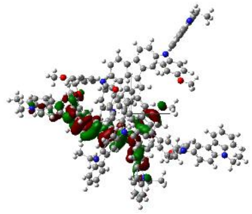	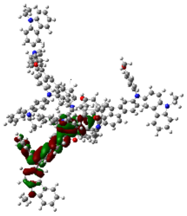	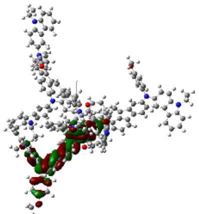
HOMO-1	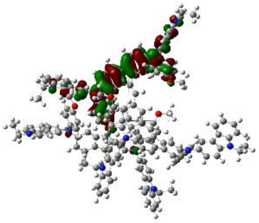	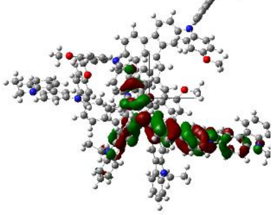	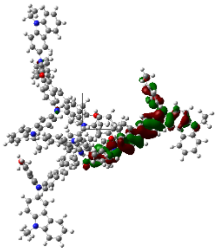	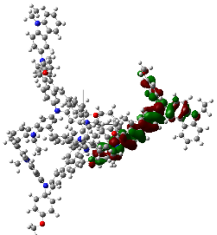
HOMO-2	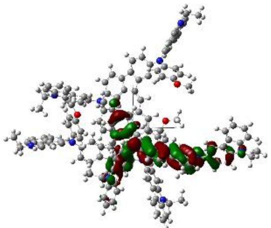	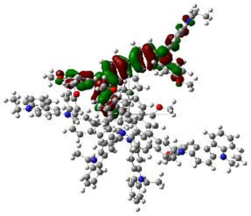	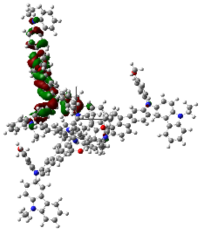	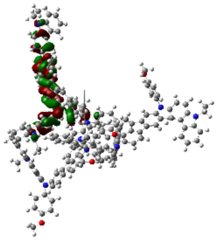

Table S7. Parameters of electronic excitations (transition energy ΔE_n and corresponding oscillator strength f_n) simulated using semiempirical TD method (for singlets).

Compound	$\Delta E_1(S_0 \rightarrow S_1)$, eV	f_1	$\Delta E_2(S_0 \rightarrow S_2)$, eV	f_2
**V1322a**	1.69	0.0189	1.81	0.0235
**V1322b**	1.57	0.0179	1.61	0.0213
**V1387a**	2.02	0.0348	2.06	0.0315
**V1387b**	2.11	0.0238	2.11	0.0568
**V1391a**	2.18	0.0402	2.19	0.0576
**V1391b**	2.19	0.0450	2.22	0.0439
**V1424a**	1.92	0.0213	2.01	0.0338
**V1424b**	1.89	0.0262	1.93	0.0224
**V1388a**	2.01	0.0384	2.07	0.0187
**V1388b**	2.02	0.0361	2.08	0.0129
**V1389a**	2.00	0.0332	2.06	0.0277
**V1389b**	2.00	0.0334	2.06	0.0318

References

[1] R. Send, I. Bruder, H. Wonneberger, A. Michaela, V. Getautis, M. Daskeviciene, T. Malinauskas. (2015). *Hole-transport materials for organic solar cells or organic optical sensors* (Germany, Patent No. WO2015161989A1. WIPO (PCT). https://patents.google.com/patent/WO2015161989A1/en

[2] T. H. Schloemer, T. S. Gehan, J. A. Christians, D. G. Mitchell, A. Dixon, Z. Li, K. Zhu, J. J. Berry, J. M. Luther, A. Sellinger, *ACS Energy Lett.* **2019**, *4*, 473.
